# Diffusion MRI signal cumulants and hepatocyte microstructure at fixed diffusion time: Insights from simulations, 9.4T imaging, and histology

**DOI:** 10.1002/mrm.29174

**Published:** 2022-02-18

**Authors:** Francesco Grussu, Kinga Bernatowicz, Irene Casanova‐Salas, Natalia Castro, Paolo Nuciforo, Joaquin Mateo, Ignasi Barba, Raquel Perez‐Lopez

**Affiliations:** ^1^ Radiomics Group Vall d’Hebron Institute of Oncology Vall d’Hebron Barcelona Hospital Campus Barcelona Spain; ^2^ Prostate Cancer Translational Research Group Vall d’Hebron Institute of Oncology Vall d’Hebron Barcelona Hospital Campus Barcelona Spain; ^3^ Molecular Oncology Group Vall d’Hebron Institute of Oncology Vall d’Hebron Barcelona Hospital Campus Barcelona Spain; ^4^ NMR Lab Vall d’Hebron Institute of Oncology Vall d’Hebron Barcelona Hospital Campus Barcelona Spain; ^5^ Department of Radiology Hospital Universitari Vall d’Hebron Barcelona Spain

**Keywords:** diffusion MRI, hepatocyte, histology, liver, microstructure, Monte Carlo simulations

## Abstract

**Purpose:**

Relationships between diffusion‐weighted MRI signals and hepatocyte microstructure were investigated to inform liver diffusion MRI modeling, focusing on the following question: *Can cell size and diffusivity be estimated at fixed diffusion time, realistic SNR, and negligible contribution from extracellular/extravascular water and exchange?*

**Methods:**

Monte Carlo simulations were performed within synthetic hepatocytes for varying cell size/diffusivity L/$D_{0}$, and clinical protocols (single diffusion encoding; maximum b‐value: {1000, 1500, 2000} s/mm^2^; 5 unique gradient duration/separation pairs; SNR = {∞, 100, 80, 40, 20}), accounting for heterogeneity in ${(D}_{0},L)$ and perfusion contamination. Diffusion (D) and kurtosis (K) coefficients were calculated, and relationships between $\left(D_{0},L\right)$ and (D,K) were visualized. Functions mapping (D,K) to $\left(D_{0},L\right)$ were computed to predict unseen $\left(D_{0},L\right)$ values, tested for their ability to classify discrete cell‐size contrasts, and deployed on 9.4T ex vivo MRI‐histology data of fixed mouse livers

**Results:**

Relationships between (D,K) and $\left(D_{0},L\right)$ are complex and depend on the diffusion encoding. Functions mapping $\left(D,K\right)$ to $\left(D_{0},L\right)$ captures salient characteristics of $D_{0}\left(D,K\right)$ and L(D,K) dependencies. Mappings are not always accurate, but they enable just under 70% accuracy in a three‐class cell‐size classification task (for SNR = 20, $b_{\max}$ = 1500 s/mm^2^, δ = 20 ms, and Δ = 75 ms). MRI detects cell‐size contrasts in the mouse livers that are confirmed by histology, but overestimates the largest cell sizes.

**Conclusion:**

Salient information about liver cell size and diffusivity may be retrieved from minimal diffusion encodings at fixed diffusion time, in experimental conditions and pathological scenarios for which extracellular, extravascular water and exchange are negligible.

## 1 INTRODUCTION

Diffusion‐weighted (DW) MRI relies on the self‐diffusion of water residing in biological tissues to probe cellular microarchitecture. In classical pulsed gradient spin echo,^1^, ^2^ two diffusion gradients sensitize the acquisition to diffusion. The first gradient tags spin phases according to spatial position, whereas the second one, played out after a certain interval (known as diffusion time), cancels such tags for stationary spins. In the presence of diffusion, water molecules change their position during the diffusion time, and the tag removal is incomplete. This leads to MRI signal attenuation, which carries a signature of tissue microstructure.^3^

Model‐based methods offer practical solutions to the estimation of microenvironment properties from MRI by adopting geometric models of microstructure.^4^ This leads to tractable expressions that parametrize the signal as a function of sequence and microstructural parameters.^5^ So far, model‐based methods have found several clinical applications,^6^, ^7^, ^8^, ^9^, ^10^, ^11^, ^12^, ^13^ in spite of potential biases occurring as a result of modeling oversimplifications.^14^, ^15^ Modeling has focused on neural^16^, ^17^, ^18^, ^19^, ^20^, ^21^, ^22^ and prostate^23^, ^24^, ^25^ tissue characterization, as well on cell‐size measurements,^26^, ^27^, ^28^, ^29^ relevant in oncology. However, less attention has been paid to other organs, such as the liver.^30^, ^31^, ^32^, ^33^ Biologically specific DW MRI methods are urgently required in liver diseases, such as liver cancer, a leading cause of cancer‐related death.^34^ Liver cancer (either primary or metastatic^35^) shows a variety of microstructural characteristics. Quantitative liver MRI methods offer sensitivity to cancer pathology,^36^ but still fail to distinguish key pathological differences (e.g., substitution of either sinusoidal endothelial cells or liver hepatocytes by neoplastic cells^35^, ^37^). There is a pressing need for new clinically viable liver MRI readouts; these could help reduce the use of invasive biopsies, which sparsely sample the tissue, are prone to false negatives, and can result in complications for the patient,^38^ and could support diagnosis and treatment selection.

A key step in diffusion MRI development is the identification of microstructural features that can be estimated from clinical‐like (i.e., intermediate b‐values and limited scan time) measurements.^5^ To our knowledge, such a characterization for hepatocytes, which account for up to 85% of liver volume,^39^ is still lacking. Here we considered realistic hepatocyte sizes and diffusion protocols that could be feasible in the clinic (single diffusion encoding, maximum b‐value up to 2000 s/mm^2^, fixed diffusion time with gradient separation/duration Δ/δ in the range of [25; 75] ms and [10; 40] ms, SNR as low as 20 at *b* = 0). Through Monte Carlo simulations and co‐localized 9.4T ex vivo MRI and histology of fixed mouse livers, we specifically investigated the following question: *Can cell size and diffusivity be estimated from signal cumulants at fixed diffusion time and realistic SNR, under the assumption of negligible contributions from extracellular/extravascular water and water exchange?* While experiments performed at varying diffusion times are ideal for cell‐size measurement,^26^, ^27^, ^28^, ^29^, ^33^ techniques providing summary cell‐size indices with minimal acquisitions have the potential of bringing quantitative MRI one step closer to the clinic.

## 2 METHODS

We simulated intracellular signals at fixed diffusion time and processed them to estimate cell size L and cell diffusivity $D_{0}$. The approach was also tested on 9.4T ex vivo MRI scans of fixed mouse livers. All analysis code is made available ( https://github.com/fragrussu/MChepato ), and was executed on two Ubuntu 20.04.2 machines (18‐core, 3.00‐GHz Intel® Core i9‐10980XE CPU).

### 2.1 Cell generation

We simulated hepatocytes (polygonal cells^39^) by perturbing regular prisms with square/pentagonal/hexagonal bases. Prisms were described by triangular meshes and featured a characteristic length *L* (base‐to‐base height and diameter of the circumcircle relative to each base). We considered 33 values of *L* in [11; 60] μm (increment: 1.5 μm), obtaining S= 15 unique cell shapes for each value of *L*. The S cells at fixed *L* were obtained by perturbing each prism base shape 5 times, displacing vertices at random (displacements drawn from a normal distribution, σ = 0.1*L*). The range for *L* covers sizes seen in healthy mammal livers (e.g., 20–30 μm in humans,^39^ 30–40 μm in mice^40^) and in pathology (e.g., swollen hepatocytes in steatosis^37^; hepatocyte substitution by smaller cancer cells^41^). Supporting Information Figure S1 shows synthetic cells.

### 2.2 Intracellular spin dynamics

We generated random walks with the MCDC simulator,^42^ distributing N = 1000 spins uniformly inside each cell (elastic reflection at walls; impermeable walls). We simulated $T_{s}$ = 140 ms (3000 steps) and varied the intrinsic cell diffusivity $D_{0}$ in [0.20; 2.40] $\frac{\mu m^{2}}{\mathrm{ms}}$ (45 values; increment: 0.05 $\frac{\mu m^{2}}{\mathrm{ms}}$).

### 2.3 Magnetic resonance imaging signal synthesis

For each fixed (*D*_0_, *L*) value, we pooled together spin trajectories $r_{n,s,k}\left(t\right)$ simulated within a neighborhood Ω of $\left(D_{0},\;L\right)$ (i.e., $\begin{array}{c} \Omega \left(D_{0},\;L\right)\;≜\;\left\{D_{0}‐0.10,\;\;D_{0}‐0.05,\;\;D_{0},\;\;D_{0}+0.05,\;\;D_{0}+0.10\right\}\\ \;\frac{\mu m^{2}}{\mathrm{ms}}\;\times \;\left\{L‐3.0,\;L‐1.5,\;L,\;L+1.5,\;L+3.0\;\right\}\;\mu m) \end{array}$. This introduces heterogeneity expected in realistic voxels,^15^, ^43^ leading to 1189 $\left(D_{0},\;L\right)$ pairs. Above, n = 1, …, N is the index of a spin within a cell; s= 1, …, S is the cell‐shape index for fixed cell size; and k=1,⋯,Kenumerates the elements of $\Omega \left(D_{0},\;L\right)$, with $K=dim\left(\Omega \left(D_{0},\;L\right)\right)$. For MRI signal synthesis, we considered single diffusion encoding^2^ gradient waveforms G(t), with five unique clinically realistic gradient duration/separation δ/Δ ([10 ms, 50 ms], [20 ms, 25 ms], [20 ms, 50 ms], [20 ms, 75 ms], and [40 ms, 50 ms]). For any fixed (δ, Δ), we synthesized measurements corresponding to seven nonzero b‐values, uniformly spaced in ($b_{\min}$;$b_{\max}$), where $b_{\min}$ = 100 s mm^–2^, a value used to suppress intravoxel incoherent motion (IVIM)–like components^44^, ^45^; and $b_{\max}$ = (1000, 1500, 2000) s mm^–2^, as the volume‐weighted^46^ sum:

(1) $$\begin{array}{c} s_{\mathrm{intra}}\left(D_{0},\;L\right)=\;\sum_{k=1}^{K}\sum_{s=1}^{S}\;\;\frac{L_{s,k}^{3}}{\sum_{v=1}^{K}\sum_{u=1}^{S}L_{u,v}^{3}}\\ \;\;\;\left|\;\;\frac{1}{N}\sum_{n=1}^{N}e^{‐j\;\gamma \;\Delta t\;\sum_{t=0}^{T_{s}}\;G^{T}\left(t\right)\;r_{n,s,k}\left(t\right)\;}\;\right|. \end{array}$$

For each b‐value, we generated signals for three mutually orthogonal gradients (as common in liver MRI^44^, ^45^), averaged them, and introduced random slow‐flow (intravoxel incoherent motion, or IVIM)^44^, ^47^ contamination as follows:

(2) $$s\;\;=\;\;f\;e^{‐b\;D_{v}}+\left(1‐f\right)\;s_{\mathrm{intra}}.$$

In this equation, 0.05≤f≤0.50 controls the IVIM contamination, and $15\;\frac{\mu m^{2}}{\mathrm{ms}}\leq D_{v}\leq 60\;\frac{\mu m^{2}}{\mathrm{ms}}$.^44^, ^45^ Rician noise was injected at an SNR of (∞, 100, 80, 40, 20), where ∞ denotes no noise added; SNR=1/σ, $\sigma^{2}$ denotes the noise variance.

### 2.4 Analysis

We estimated apparent diffusion/kurtosis coefficients D/K for any $\left(D_{0},\;L\right)$, diffusion protocol, and SNR by fitting^5^, ^48^

(3) $$\ln\left(s\right)\;=\;\ln\left(s_{0}\right)\;‐bD\;+\;\;\frac{1}{6}\;K\;{\left(bD\right)}^{2}$$

through constrained nonlinear least‐squares fitting initialized by linear fitting ($0\leq \;s_{0}\leq 1$; $0\;\frac{\mu m^{2}}{\mathrm{ms}}\leq \;D\leq 2.4\;\frac{\mu m^{2}}{\mathrm{ms}}$; ‐5≤K≤10). The value of $s_{0}$ is the non‐DW signal.

We tested whether $\left(D_{0},\;L\right)$ can be estimated from D and K when the contribution of extracellular, extravascular water and transcytolemmal water exchange are negligible. To this end, we related (D,K) to $\left(D_{0},\;L\right)$ using color‐coded scatter plots, and studied paired $\left(D,\;K\right)\to \;D_{0}$ and $\left(D,\;K\right)\to \;L$ observations estimating smooth function ($D_{0}\left(D,\;K\right)$, $L\left(D,\;K\right)$) mapping $\left(D,\;K\right)$ to $\left(D_{0},\;L\right)$ at a fixed protocol and SNR. The estimation was based on the following polynomial functions:

(4) $$\begin{array}{c} m=\;a_{0}+a_{1}D+a_{2}K{+a_{3}DK+a}_{4}D^{2}+\;a_{5}K^{2}+\;a_{6}D^{2}K\\ +a_{7}DK^{2}+\;a_{8}D^{3}+a_{9}K^{3}, \end{array}$$

where *m* indicates $D_{0}$ and L in turn. We refer to the estimation of $D_{0}\left(D,K\right)$ and L(D,K) via Equation 4 as *PolyMap*. Coefficients *a_i_* were estimated on 700 randomly selected $\left(D_{0},\;L\right)$ training pairs out of 1189, and then deployed to predict the 489 unseen $\left(D_{0},\;L\right)$ values. We assessed the quality of the prediction by visualizing errors against ground‐truth values. For reference, *PolyMap* was compared with fitting of a biophysical model of the intracellular DW signal (*SigFit* estimation) as follows:

(5) $$s\approx s_{0}\exp\left(‐\;b\;\left(c_{0\;}\frac{L^{4}}{\;D_{0}\;\delta \;\left(\Delta \;‐\;\frac{\delta }{3}\right)\;\;}‐c_{1}\frac{L^{6}}{\;{D_{0}}^{2}\;\delta^{2}\;\left(\Delta \;‐\;\frac{\delta }{3}\right)\;\;}\right)\right)\;\;.$$

Equation 5 relies on an approximate expression of the apparent diffusion coefficient for spins diffusing in a bounded medium (wide‐pulse limit).^49^, ^50^, ^51^ The values of $c_{0}$ and $c_{1}$ are constants that depend on the geometry: Analysis of intracellular diffusion coefficients from 400 unique coefficients $\left(D_{0},\;L,\;\Delta ,\delta \right)$ provides $c_{0}\approx \;1.342∙10^{‐3}$, $c_{1}\approx \;1.259∙10^{‐5}$ for our synthetic cells. Note that $D_{0}$ and L in Equation 5 are fitted jointly to sets of signal measurements performed at varying b‐value (but fixed Δ and δ). This implies that we do not get a single number for the apparent diffusion coefficient value first, and derive $D_{0}$ and L from it afterwards.

Finally, we tested whether it is possible to resolve cell‐size contrasts with the minimal protocols considered here, being that $D_{0}$ and L are difficult to disentangle. We discretized L as small (L≤28μm), medium (28μm<L≤42μm), large (L>42μm), and fitted a multinomial logistic regression model (Python statsmodels) in the same form of Equation 4 ($m=\left\{0,\;1,\;2\right\}$: discretised L). The model was fitted to the training set for all diffusion protocols and SNR = 20, and deployed on the validation set. We calculated classification accuracy and estimated 95% accuracy ranges compatible with chance by training on 1000 random permutations of the m labels.

### 2.5 Magnetic resonance imaging ‐ histology comparison

Two formalin‐fixed NOD.Cg‐Prkdc^scid^ IL2rg^tm1WjI^/SzJ mouse livers from an approved, ongoing study (wild‐type [WT] and patient‐derived xenograft (PDX), subcutaneous implantation of prostate cancer bone biopsy) were scanned in phosphate‐buffered saline on a 9.4T Bruker Avance system (room temperature) to test whether our approach can detect histologically meaningful cell‐size differences due to pathology. The DW spin‐echo scans (Δ = 30 ms; δ = 10 ms; TE = 45 ms; TR = 2700 ms; 10 b‐values in [0; 4500] s/mm^2^; two slices, 1‐mm thick; 349 × 273 μm^2^ resolution) were acquired and preprocessed.^52^, ^53^, ^54^ Images acquired at *b* > 1700 s/mm^2^ (i.e., with negligible phosphate‐buffered saline contamination) were analyzed with *PolyMap* and *SigFit*. For *PolyMap* computation, the $\left(D,K\right)\to \left(D_{0},L\right)$ mapping was learned on signals synthesized for the specific protocol used ex vivo, and corrupted at an SNR equal to the sample median SNR at *b* = 0, estimated through Marchenko and Pastur principal component analysis.^52^, ^55^ *SigFit* fitting was instead performed by either (1) estimating jointly $D_{0}$ and L, or (2) fixing $D_{0}$ to {0.5, 0.75, 1.0, 1.25, 1.50} $\frac{\mu m^{2}}{\mathrm{ms}}$ in turn to all voxels and then estimating L, as in some model‐based approaches.^26^

One 4‐μm‐thick histological section was obtained for each MRI slice, stained with hematoxylin and eosin, and digitized (Hamamatsu C9600‐12 scanner; resolution: 0.227 μm). Cells were segmented with QuPath,^56^ obtaining cell‐wise diameters l. These were analyzed within patches matching the in‐plane MRI resolution, deriving per‐patch histological cell size

(6) $$L_{\mathrm{histo}}={\left(\frac{\;<l^{7}>\;}{<l^{3}>}\right)}^{\frac{1}{4}}.$$

Equation 6 is justified by noting that the total intracellular MRI signal $s_{\mathrm{intra}}$ is approximately proportional to $\frac{<l^{7}>\;}{<l^{3}>}$, being that $s_{\mathrm{intra}}=\;\frac{\;<\;l^{3}s\left(l\right)\;>\;}{<\;l^{3}\;>}$ is the volume‐weighted sum^57^ of individual cell signals and that $s\left(l\right)\;\approx \;e^{‐\alpha l^{4}}\;\approx \;1‐\alpha l^{4}$,^49^ implying that $s_{\mathrm{intra}}\approx \;1‐\alpha \frac{\;<l^{7}>\;}{<l^{3}>}.L_{\mathrm{histo}}$ was warped to MRI (symmetric diffeomorphic registration^58^ of specimens’ manual outlines), and metric distributions were evaluated.

## 3 RESULTS

The computation time required to process one MRI protocol was approximately 700 seconds for each $\left(D_{0},L\right)$ pair on one CPU.

**FIGURE 1:**
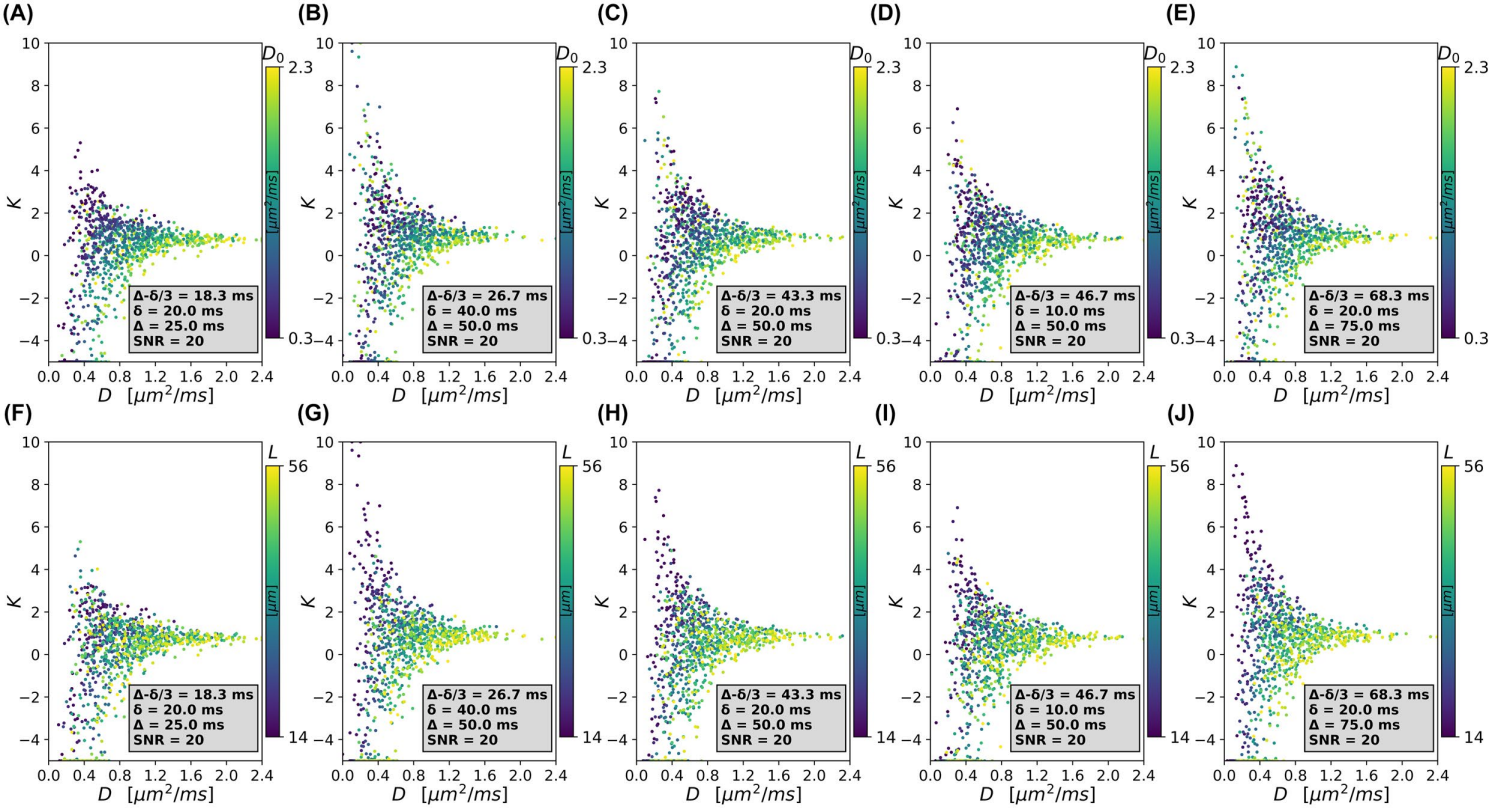
Scatter plots of (D,K) color‐coded by the underlying average intrinsic cell diffusivity $D_{0}$ (top, A‐E) and cell size L (bottom, F‐J), as obtained when noise is added to the synthetic MRI signals at an SNR at *b* = 0 of 20. From left to right: Different diffusion times (δ/Δ = 20/25 ms in [A] and [F]; δ/Δ = 40/50 ms in [B] and [G]; δ/Δ = 20/50 ms in [C] and [H]; δ/Δ = 10/50 ms in [D] and [I]; and δ/Δ = 20/75 ms in [E] and [J]). The figure refers to a minimum/maximum protocol b‐value of b = 100/2000 s/mm^2^. Noise‐free intracellular diffusion‐weighted (DW) signals are contaminated by intravoxel incoherent motion (IVIM)–like partial volume

Plots in Figure 1 scatter D against K. Points in the (D,K) plane correspond to a unique ${(D}_{0},L)$ combination, and are colored according to $D_{0}$ (top) and L (bottom). The figure refers to maximum b‐value of 2000 s/mm^2^, SNR = 20, and multiple combinations of (δ,Δ). The values of D and K exhibit a wide range of variation (e.g., negative K values are seen), depending on protocol δ and Δ. Nonetheless, a trend in the $D_{0}$/L coloring can be seen (more apparent as SNR increases). In absence of noise, a non‐monotonic relationship between (D,K)and both $D_{0}$ and L is seen, with points distributed according to complex patterns in the (D,K) domain (Supporting Information Figure S2). For some specific combinations of (D,K), no experimental points are observed. The position of the points in the (D,K) plane changes depending on (δ,Δ). For example, when $b_{\max}$ = 2000 s/mm^2^, the median/95% range of D are 0.76/[0.27; 1.57] $\frac{\mu m^{2}}{\mathrm{ms}}$ for δ/Δ = 20/25 ms and 0.52 [0.16; 1.20] $\frac{\mu m^{2}}{\mathrm{ms}}$ for δ/Δ = 20/75 ms. For the same gradient timings, median/95% ranges of K are 0.44 [0.29; 1.53] and 0.40 [0.07; 2.99]. Results for smaller maximum b‐values follow similar trends, although numerical values of (D,K) depend on $b_{\max}$, both in absence or presence of noise (e.g., SNR = 20) (Supporting Information Figure S3 for $b_{\max}$ = 1000 s/mm^2^). In absence of noise and when $b_{\max}\;$ = 1000 s/mm^2^, the median/95% range of D values are 0.79 [0.28; 1.61] $\frac{\mu m^{2}}{\mathrm{ms}}$ for δ/Δ = 20/25 ms and 0.55 [0.17; 1.25] $\frac{\mu m^{2}}{\mathrm{ms}}$ for δ/Δ = 20/75 ms, whereas it is 0.58 [0.29; 3.12] and 0.78 [0.14; 6.33] for K, larger than what is seen when $b_{\max}\;$ = 2000 s/mm^2^.

**FIGURE 2:**
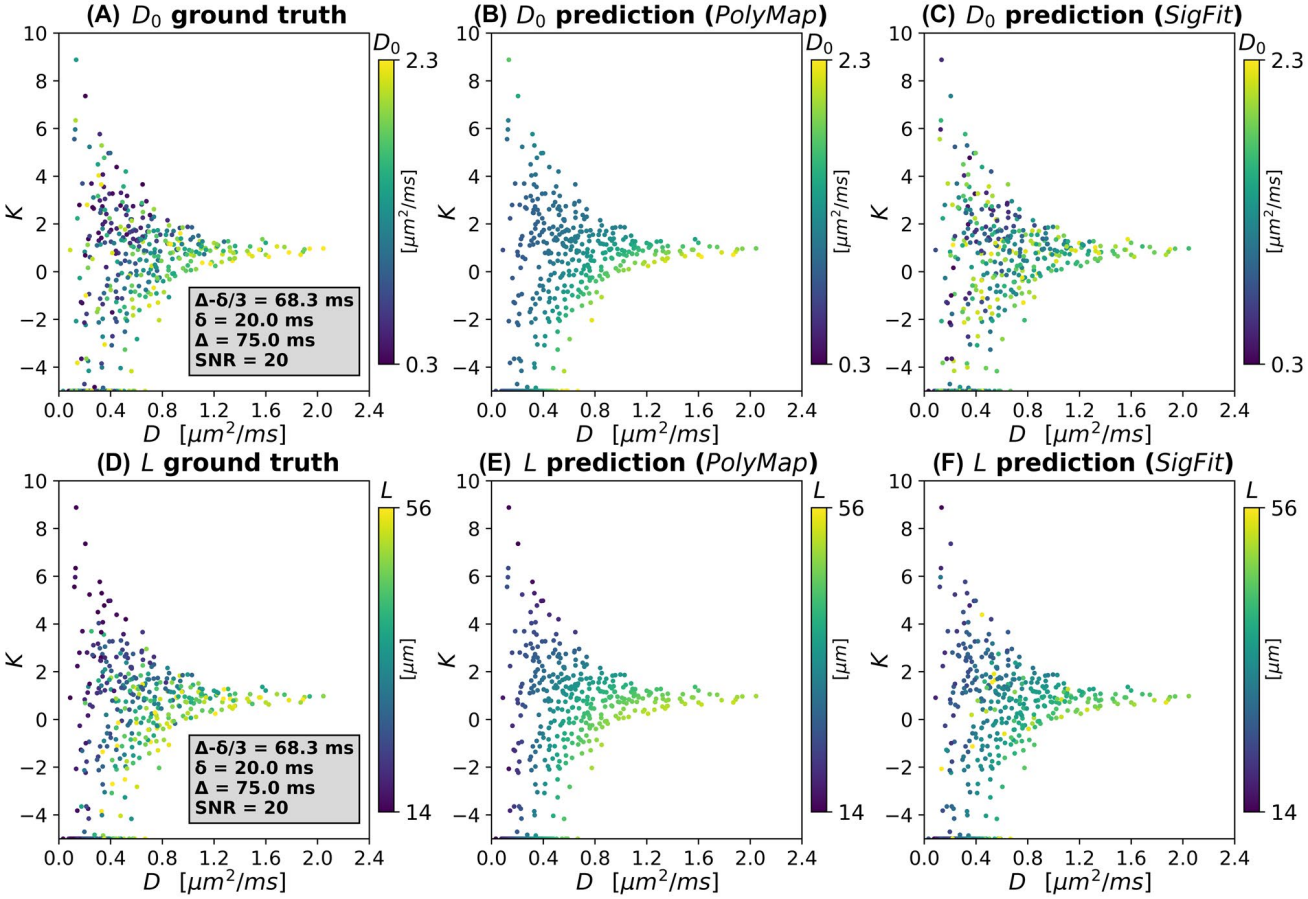
Examples of predictions of intrinsic cell diffusivity $D_{0}$ and cell size L on the validation set. (A–C) Scatter plots colored by cell diffusivity $D_{0}$. (D–F) Scatter plots colored by cell size L. Left: Signal cumulants $\left(D,K\right)$ at fixed diffusion time colored by underlying ground truth $D_{0}$ and L. Middle: Signal cumulants $\left(D,K\right)$ at fixed diffusion time colored by predictions of $D_{0}$ and L as obtained with *PolyMap*. Right: Signal cumulants $\left(D,K\right)$ at fixed diffusion time colored by predictions of $D_{0}$ and L as obtained with *SigFit*. The figure refers to the case when the minimum/maximum protocol *b*‐values is equal to b = 100/2000 s/mm^2^ and the diffusion gradient duration/separation is δ = 20 ms/Δ = 75 ms, for SNR of 20 and in presence of IVIM contamination

Figure 2 shows (D,K) scatter plots color‐coded by $D_{0}$ and L for observations belonging to the validation set ($b_{\max}$ = 2000 s/mm^2^, δ = 20 ms, Δ = 75 ms, SNR = 20). It also shows $D_{0}$ and L predicted in correspondence of the same (D,K) values with both *PolyMap* and *SigFit*. Similar plots for the noise‐free case and different protocols (e.g., $b_{\max}$ = 1000 s/mm^2^, δ = 20 ms, Δ = 25 ms) are reported in Supporting Information Figures S4 and S5. Overall, *PolyMap* does not necessarily predict accurately the values of $D_{0}$ and L on unseen data, especially at high noise level. Nonetheless, it captures the salient characteristics of the $D_{0}\left(D,K\right)$ and L(D,K) relationships, which appear unique to each diffusion‐encoding protocols and SNR. The *SigFit* estimation also captures $D_{0}$ and L contrasts, although predictions are less smooth than those from *PolyMap*.

**FIGURE 3:**
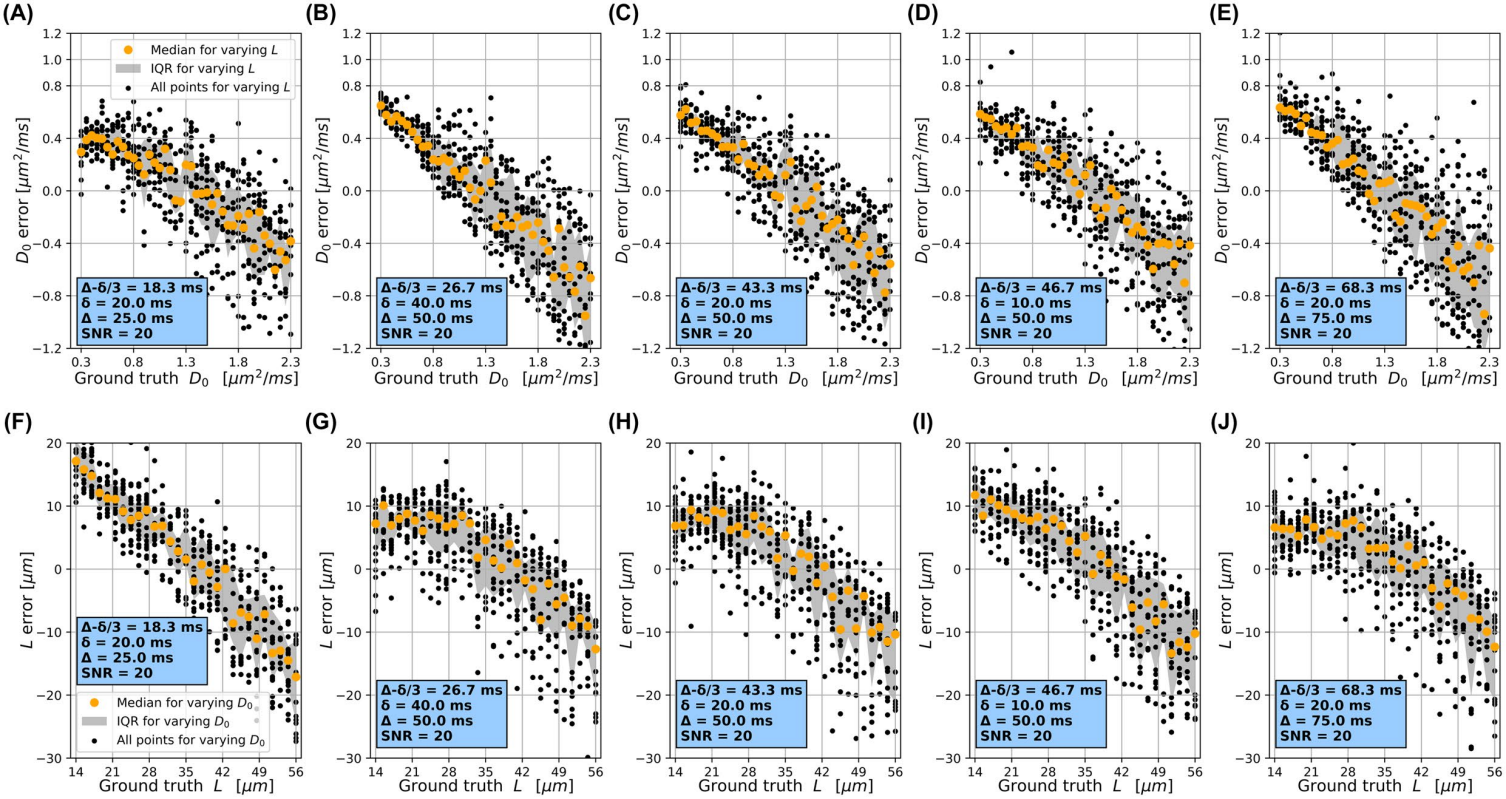
$D_{0}$ and L prediction errors for the *PolyMap* estimation method, scattered against ground‐truth values of $D_{0}$ and L, for different diffusion gradient timings at a fixed maximum b‐value of 2000 s/mm^2^ and SNR of 20. From left to right: Different gradient timings (δ/Δ = 20/25 ms in [A] and [F]; δ/Δ = 40/50 ms in [B] and [G]; δ/Δ = 20/50 ms in [C] and [H]; δ/Δ = 10/50 ms in [D] and [I]; and δ/Δ = 20/75 ms in [E] and [J]). Plots on top (A‐E) refer to $D_{0}$; plots on the bottom (F‐J) refer to L. For each fixed value of $D_{0}$ (on top, or L to the bottom), median errors with interquartile ranges for varying L (on top, or varying $D_{0}$ to the bottom) are also reported

Figure 3 plots *PolyMap* $D_{0}$ and L prediction errors (prediction – ground truth) against ground‐truth $D_{0}$ and L for different protocols ($b_{\max}$ = 2000 s/mm^2^, SNR = 20). The same plots corresponding to *SigFit* are reported in Supporting Information Figure S6. Further *PolyMap* and *SigFit* prediction errors for the noise‐free case and for $b_{\max}$ = 1000 s/mm^2^, SNR = 20, are included in Supporting Information Figures S7 and S8. The charts reveal that $D_{0}$ and L are overestimated/underestimated at the lower/upper end of their ranges. This trend is observed for different gradient timings and in absence of noise, although to a lesser extent. Higher SNR and longer diffusion times lead to smaller errors. The $D_{0}/L$ *PolyMap* errors are slightly smaller/larger than those from *SigFit*.

Table 1 reports validation‐set accuracies for the cell‐size classification task. Accuracy values can be as high as almost 70%, such as when $b_{\max}$ = 1500 s/mm^2^, δ = 20 ms, and Δ = 75 ms, corresponding to 86%, 46%, and 61% correctly classified small, medium, and large cells. Accuracies are above accuracy ranges compatible with chance.

**TABLE 1:** Accuracies obtained on the validation set for the three‐class cell‐size classification task performed using multinomial logistic regression at SNR = 20 and for all diffusion‐encoding protocols

		*b*_max_ = 1000 s/mm^2^	*b*_max_ = 1500 s/mm^2^	*b*_max_ = 2000 s/mm^2^
δ = 20 ms, Δ = 25 ms, Δ – δ/3 = 18.3 ms	Accuracy	0.54	0.54	0.55
	95% random interval	[0.23; 0.42]	[0.22; 0.43]	[0.22; 0.43]
δ = 40 ms, Δ = 50 ms, Δ – δ/3 = 36.7 ms	Accuracy	0.60	0.61	0.61
	95% random interval	[0.21; 0.46]	[0.21; 0.46]	[0.19; 0.48]
δ = 20 ms, Δ = 50 ms, Δ – δ/3 = 43.3 ms	Accuracy	0.58	0.56	0.60
	95% random interval	[0.21; 0.45]	[0.21; 0.45]	[0.20; 0.46]
δ = 10 ms, Δ = 50 ms, Δ – δ/3 = 46.7 ms	Accuracy	0.57	0.56	0.54
	95% random interval	[0.22; 0.43]	[0.21; 0.45]	[0.23; 0.42]
δ = 20 ms, Δ = 75 ms, Δ – δ/3 = 68.3 ms	Accuracy	0.61	0.67	0.63
	95% random interval	[0.21; 0.45]	[0.19; 0.47]	[0.20; 0.48]

Note:

The table also includes the estimated 95% interval of accuracies that can be expected due to chance. An accuracy of 1.00 implies that all validation observations have been correctly classified; an accuracy of 0.00 implies instead that none have been correctly classified.

**FIGURE 4:**
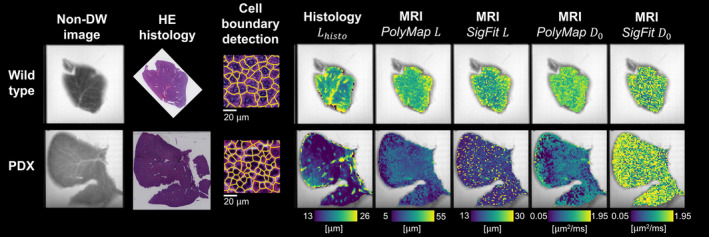
Estimation of intrinsic cell diffusivity $D_{0}$ and cell size L from the 9.4T ex vivo MRI scans of fixed mouse livers, with co‐localized hematoxylin and eosin (HE) histology. Top: Wild‐type (WT) case. Bottom: Patient‐derived xenograft (PDX) case (subcutaneous implantation of bone biopsy from metastatic prostate cancer). From left to right: Non‐DW image; co‐localized HE; example of cell segmentation on HE; histology‐derived cell size index $L_{\mathrm{histo}}$; MRI cell size L estimates through *PolyMap* and *SigFit* estimation; MRI cell diffusivity $D_{0}$ estimates through *PolyMap* and *SigFit* estimation

Figure 4 reports MRI histology results. Unlike the WT, the PDX features widespread infiltration of smaller cells (likely leukocytes) in between larger hepatocytes. This leads to between‐sample $L_{\mathrm{histo}}$ contrast ($L_{\mathrm{histo}}$ higher in WT than PDX), replicated in *PolyMap* and *SigFit* L. The value of $D_{0}$ is lower in PDX than WT in *PolyMap*, whereas no $D_{0}$ differences are seen for $D_{0}$ *SigFit*. The *SigFit* metrics feature salt‐and‐pepper variations and are less smooth than *PolyMap*. Distributions (Supporting Information Table S1) confirm that L agrees well with $L_{\mathrm{histo}}$ for both *PolyMap* and *SigFit* in PDX. In WT, L is larger than $L_{\mathrm{histo}}$, especially for *PolyMap*. The value of *SigFit* $D_{0}$ is more variable than *PolyMap* $D_{0}$ in both specimens. Supporting Information Figure S9 reports signal predictions from fitted parameters for both WT and PDX livers, highlighting that both *SigFit* and *PolyMap* provide good quality of fit. Supporting Information Figure S10 reports alternative *SigFit* cell‐size estimates L obtained when $D_{0}$ is fixed to a specific value for all voxels, and not fitted. The value of L obtained at fixed $D_{0}$ is highly dependent on the value used for $D_{0}$: For some specific values, the between‐sample cell‐size contrast is even reversed, with larger L in the PDX than in the WT, a finding that disagrees with histology.

## 4 DISCUSSION

### 4.1 Summary and key findings

We performed simulations to relate DW signal features (i.e., apparent diffusion/kurtosis coefficients, D and K) to cell microstructure (cell diffusivity/size, $D_{0}$ and L) at fixed diffusion time, under the hypothesis of negligible sensitivity to extracellular/extravascular water and exchange. We also used cell‐size mappings learned from simulations on 9.4T ex vivo MRI of fixed mouse livers, comparing results to histology. Our work is motived by the fact that estimating summary cell‐size contrasts with minimal protocols may be useful in hospital settings, where scan time is limited and the latest technologies are not available.

Our main finding is that D and K offer sensitivity to $D_{0}$ and L even when computed at realistic SNR levels, so it appears feasible to establish a mapping $\left(D,K\right)\;\to \;\left(D_{0},L\right)$. Although the mapping does not estimate accurately $D_{0}$ and L for the studied range, it captures salient cell‐size contrasts at fixed diffusion time. On the 9.4T MRI data, $\left(D,K\right)\;\to \;\left(D_{0},L\right)\;$ mappings provide cell‐size contrasts that are confirmed by histology, but overestimate L, especially for larger cells. The overestimation of L is less strong when this is estimated through biophysical models of restricted diffusion, which were considered as a potential alternative to $\left(D,K\right)\;\to \;\left(D_{0},L\right)\;$ mappings, at the price of more variable parametric maps (especially $D_{0}$).

### 4.2 Simulations

We used state‐of‐the‐art Monte Carlo simulations^42^ to study DW MRI protocols that could be implemented in the clinic (i.e., intermediate b‐values, fixed diffusion time, short scan time). Our results demonstrate that associations between D and K from such protocols and cell diffusivity $D_{0}$ and size L, exist. The relationship is complex and non‐monotonic, with relatively small changes in $D_{0}$ and L causing large variations of K and D. This may imply that biophysical liver models may benefit from intra‐compartmental kurtosis in the hepatocyte compartment, to better capture departures from Gaussian diffusion.

We also used paired (D,K) and $\left(D_{0},L\right)$ to compute polynomial functions that estimate $D_{0}$ and L from D and K (*PolyMap*). Such functions offer sensitivity to the underlying $D_{0}$ and L, even when computed on noisy data (SNR = 20). Although the estimates are not accurate for the smallest and largest values of $D_{0}$ and L, they may suffice to characterise large cell‐size variations, such as distinguishing discrete cell‐size contrasts, as demonstrated through multinomial logistic regression. For reference, *PolyMap* was compared with fitting $D_{0}$ and L based on a biophysical model of the DW signal (*SigFit* approach). Results from *SigFit* are in line with those from *PolyMap*. Although *SigFit* enables slightly more accurate L estimation than *PolyMap* for the low‐intermediate values L, *PolyMap* L estimates are closer to ground‐truth values for L of the order of 40 µm to 50 µm, plausible in pathological processes such as hepatocyte ballooning.^59^ Moreover, *PolyMap* exhibits higher precision (smoother $D_{0}\left[D,K\right)]$ and L[D,K]) and better resolves $D_{0}$. These results suggest that the relative performances of *PolyMap* and *SigFit* depend on the diffusion regime, and, more importantly, that overall it may be feasible to obtain summary descriptors of cell size from clinical acquisitions at fixed diffusion time, if analyzed with appropriate techniques. Such an approach could have application in high‐risk populations, such as patients with a history of hepatitis (at risk of hepatocellular carcinoma^60^) or primary colorectal cancer (at risk of liver metastases^61^), and in contexts in which implementing rich acquisitions is not possible. Moreover, mappings $\left(D,K\right)\;\to \;\left(D_{0},L\right)$ tuned for specific diffusion encodings may help mitigate interscanner variability. In that respect, they may prove useful in the retrospective analysis of multicenter clinical data featuring a variety of acquisition protocols. Nonetheless, we remark that acquiring prospective data at varying diffusion weightings and times should be the preferred way to perform cell‐size estimation, when possible.

### 4.3 Magnetic resonance imaging and histology

We estimated $D_{0}$ and L on 9.4T ex vivo DW images of two formalin‐fixed mouse livers (one WT, one PDX), acquired at fixed diffusion time (Δ = 30 ms; δ = 10 ms). The MRI indices were related to co‐localized histological cell size $L_{\mathrm{histo}}$, confirming results from simulations. Both *PolyMap* and *SigFit* provide good signal quality of fits, suggesting that they both are good representations of the diffusion MRI signal. Moreover, they both detect diffuse cell size L alterations in the PDX liver that are confirmed by $L_{\mathrm{histo}}$, despite overestimating actual cell‐size values ($L_{\mathrm{histo}}$ is consistently lower than L from MRI). This finding agrees with the overestimation seen in simulations for ground‐truth sizes of up to 35 μm to 40 μm, and may also result, at least in part, from histological tissue shrinkage and biases from neglected extracellular/extravascular water. Nonetheless, we acknowledge that the overestimation of L is higher for *PolyMap* than for *SigFit*, especially for the WT liver. For *PolyMap*, we used a $\left(D,K\right)\;\to \;\left(D_{0},L\right)$ mapping evaluated at a single, fixed SNR. It is possible that more accurate results could be obtained learning a $\left(D,K\right)\;\to \;\left(D_{0},L\right)$ mapping for each voxel, tailored to spatially variant noise, or using more sophisticated $\left(D,K\right)\;\to \;\left(D_{0},L\right)$ mapping strategies beyond polynomial fitting (e.g., random forests).^62^ These are likely to outperform *PolyMap*, while also providing clearer biological interpretations than polynomial expansions, whose optimal degree is challenging to determine.

Notably, *PolyMap* detects PDX‐WT differences in $D_{0}$, unlike *SigFit*. While it is challenging to verify this on the type of histological data at hand (routine hematoxylin and eosin staining), we speculate that it is possible that some of these between‐sample $D_{0}$ differences may exist. Supporting Information Figure S11 provides examples of the strikingly different cellular composition characterizing the two livers. On visual inspection, hepatocytes in the WT liver contain more fat than those in the PDX. Moreover, the PDX liver is characterized by a nonspecific, lymphoma‐like process, in which cells that are much smaller than normal hepatocytes invade vascular and extravascular spaces. Such cells may feature a distinct intracellular microenvironment as compared with normal hepatocytes, resulting in per‐cell $D_{0}$ heterogeneity. Taken as a whole, these findings suggest that differences in terms of intrinsic intracellular diffusivity $D_{0}$ between the two specimens cannot be ruled out a priori. In future work, we aim to perform richer immunohistochemical analyses to gain insight into the tortuosity of the intracellular space, and thus derive histological counterparts of $D_{0}$ to confirm our MRI findings.

Regarding our 9.4T diffusion MRI acquisition, we used a maximum b‐value of 4500 s/mm^2^. This is considerably higher than in simulations, where it never exceeds 2000 s/mm^2^, as in some clinical studies.^63^ This can be justified by considering that reductions of up to three times of the average apparent diffusion coefficient can be expected when scanning fixed liver tissue, as compared with the in vivo case.^64^ Therefore, b = 4500 s/mm^2^ is expected to cause a signal attenuation somewhat comparable to approximately 1500 s/mm^2^ in vivo. Also, on the ex vivo data we perform *PolyMap* and *SigFit* analyses using a minimum b‐value of 1700 s/mm^2^. This is done to suppress partial‐volume effects with vessels and capillaries, which are filled at least in part with phosphate‐buffered saline. The diffusivity of phosphate‐buffered saline (1.8–2.0 $\frac{\mu m^{2}}{\mathrm{ms}}$) is at least 8–10 times lower than the pseudo‐diffusion coefficient of the IVIM water pool in vivo (15–60 $\frac{\mu m^{2}}{\mathrm{ms}}$), justifying the use of a minimum b‐value of 1700 s/mm^2^ against 100 s/mm^2^ as done in simulations.

We acknowledge that in this study we tested whether mappings learned on simulated MRI signals could be deployed on actual MRI measurements, performed on fixed ex vivo tissue at 9.4 T. In future work we aim to test such mappings on actual clinical MRI scans of the human liver, and investigate the performance of the approach in the presence of lower SNR, motion, and perfusion.

### 4.4 Methodological considerations

We used a simple geometric model based on perturbations of regular prisms^65^ to capture restricted diffusion. Although it sufficed to introduce variability in cell shape and to avoid overly simplistic representations (e.g., cubes), different models (e.g., meshes from histological images) could have been used. We plan to explore them in future work.

Another aspect is that our simulations focused on hepatocytes. We included heterogeneity in cell size/diffusivity, and accounted for partial volume with incoherent perfusion,^32^, ^47^, ^66^, ^67^ effectively relying on a two‐compartment model, under the hypothesis that the sensitivity to extracellular, extravascular water and transcytolemmal exchange are negligible. This assumption may be reasonable in the healthy liver, as hepatocytes are tightly packed within hepatic lobules, and account for 70%–85% of the liver volume.^39^ They are surrounded by networks of fluid‐filled conduits (sinusoidal capillaries, whose walls embed endothelial, stellate, dendritic, and Kupffer cells; and bile ducts^68^), whose signal fraction is expected to be on the order of 10%–20%.^44^ Interestingly, this two‐compartment model may capture the essence of the DW signal even in some pathological tissues, such as metastases.^69^ Nonetheless, extracellular, extravascular water may be relevant in the presence of other pathological processes, such as in fibrosis.^70^ In those cases, an additional compartment may be needed^33^: While (D,K) may still retain sensitivity to $\left(D_{0},L\right)$, they would not be specific. Finally, we neglected transcytolemmal water exchange. Known intracellular water residence times for hepatocytes and cancer cells of [40 ms; 150 ms]^33^, ^71^ imply that neglecting exchange may be reasonable in the short/intermediate diffusion times considered here. Nonetheless, further biases^72^ may be expected for longer diffusion times. Our work represents a first exploratory characterization of the main components of the liver parenchyma and in specific measurement conditions. In future work, we will generalize our analysis to more complex tissue models.

We explored relationships between (D,K) and cell microstructure $\left(D_{0},L\right)$, and tested whether information derived from Monte Carlo simulations enables a mapping $\left(D,K\right)\to \;\left(D_{0},L\right)$. Linking cumulants to microstructure is a powerful approach that has shown promise in the brain.^18^, ^21^, ^73^ Nonetheless, (D,K) depend strongly on the diffusion‐encoding protocol used for acquisition. Therefore, one would need to learn a mapping $\left(D,K\right)\to \;\left(D_{0},L\right)$ for the specific diffusion protocol at hand (i.e., δ, Δ and b‐values). Moreover, (D,K) may be difficult to measure accurately on noisy data (e.g., K can be unstable when D is low, being computed by dividing the second cumulant by D).^74^ In the future, more advanced signal‐to‐microstructure mappings will be explored (e.g., machine learning^75^, ^76^, ^77^).

Moreover, we limited our analysis to clinical single diffusion encoding with moderate b‐values. We acknowledge that more advanced encodings may provide more accurate cell‐size figures, such as combining pulsed/oscillating gradients,^33^ b‐tensor encoding,^19^, ^78^ and power law modeling.^57^ In particular, fitting biophysical models of restricted diffusion on measurements performed at varying diffusion time is likely to outperform cell‐size estimation at fixed diffusion time. However, we note that considering such protocols goes beyond the scope of this paper: Our main focus is on simple diffusion encodings at a fixed diffusion time. Our results quantify how much information on cell size can be retrieved with such minimal schemes, being these routine in hospital settings. However, when cell‐size estimation is sought in prospective studies, we recommend that diffusion protocols probe multiple diffusion times—scan time and hardware allowing.

We compared $\left(D,K\right)\to \;\left(D_{0},L\right)$ mappings (*PolyMap*) against fitting a biophysical model of intracellular restricted diffusion (*SigFit*) on protocols including a single diffusion time. We acknowledge that analyses such as *SigFit* would normally be performed on measurements performed at variable diffusion times,^26^, ^29^, ^33^ given the challenge of resolving $D_{0}$ and L. A common way to reduce the number of tissue parameter unknowns in such model‐based approaches is to fix $D_{0}$ to a specific value across all voxels, and estimate only L. While this would likely stabilize the fitting, it may lead to unphysical solutions if inappropriate values are used for $D_{0}$. This is demonstrated in Supporting Information Figure S10 (the PDX‐WT cell‐size contrast can even be reversed depending on $D_{0}$), warning against the risks of using overly simplified analytical models in conjunction with minimal diffusion encodings.

Finally, our simulated MRI protocols were based on averaging over three gradient directions, common in liver MRI,^44^, ^45^ and included seven nonzero b‐values, corresponding to a tolerable 5/10‐minute scan. Additional analyses (Supporting Information Figure S12) show that three‐direction averaging suffices to account for anisotropy, and provides D/K that are consistent with mean diffusivity/kurtosis from tensor fits^58^ on richer directional schemes.^79^ Supporting Information Figure S13 suggests that using seven nonzero b‐values may be a reasonable compromise between accuracy/precision and scan time.

## 5 CONCLUSIONS

In experimental conditions for which extracellular, extravascular signal sources and transcytolemmal exchange can be neglected, salient but potentially relevant information on liver cell size and diffusivity may be retrieved from simple diffusion encodings at a fixed diffusion time, provided that these are analyzed with appropriate computational techniques.

## DISCLOSURES

FG was supported by PREdICT, a study funded by AstraZeneca at the Vall d’Hebron Institute of Oncology (Barcelona, Spain). The authors report no conflicts. AstraZeneca was not involved in any aspect concerning this study; it has not influenced the analysis of the data, the interpretation of the results, and the decision to submit the manuscript for publication.

## Supporting information


**FIGURE S1** Synthetic hepatocytes used in this study for Monte Carlo simulations (water diffusion was simulated within such synthetic cells), obtained by perturbing the position of the vertices of triangularly meshed regular prisms. Top to bottom: Different shapes of the prism bases (square, pentagonal, hexagonal). Left to right: Different unique perturbations. The figure also illustrates L, which is equal to the base‐to‐base height as well as the diameter of the circumcircle relative to each base
**FIGURE S2** Scatter plots of (D,K) color‐coded by the underlying average intrinsic cell diffusivity $D_{0}$ (A–E) and cell size L (F–J), as obtained when no noise is added to the synthetic MRI signals. From left to right: Different diffusion times (δ/Δ = 20/25 ms in [A] and [F]; δ/Δ = 40/50 ms in [B] and [G]; δ/Δ = 20/50 ms in [C] and [H]; δ/Δ = 10/50 ms in [D] and [I]; and δ/Δ = 20/75 ms in [E] and [J]). The figure refers to a minimum/maximum protocol b‐value of b = 100/2000 s/mm^2^. Noise‐free intracellular diffusion‐weighted (DW) signals are contaminated by intravoxel incoherent motion (IVIM)–like partial volume
**FIGURE S3** Scatter plots of (D,K) color‐coded by the underlying average intrinsic cell diffusivity $D_{0}$ (A–E) and cell size L (F–J), as obtained when noise is added to the synthetic MRI signals at an SNR at *b* = 0 of 20. From left to right: Different diffusion times (δ/Δ = 20/25 ms in [A] and [F]; δ/Δ = 40/50 ms in [B] and [G]; δ/Δ = 20/50 ms in [C] and [H]; δ/Δ = 10/50 ms in [D] and [I]; and δ/Δ = 20/75 ms in [E] and [J]). The figure refers to a minimum/maximum protocol b‐value of b = 100/1000 s/mm^2^. Noise‐free intracellular DW signals are contaminated by IVIM‐like partial volume
**FIGURE S4** Examples of predictions of intrinsic cell diffusivity $D_{0}$ and cell size L on the validation set. (A–C) Scatter plots showing prediction of average intrinsic cell diffusivity $D_{0}$. (D–F) Scatter plots showing prediction of average cell size $D_{0}$. Left: Signal cumulants $\left(D,K\right)$ at fixed diffusion time colored by underlying ground‐truth $D_{0}$ and L. Middle: Signal cumulants $\left(D,K\right)$ at fixed diffusion time colored by predictions of $D_{0}$ and L as obtained with the *PolyMap* approach, which relies on using smooth functions $D_{0}\left(D,K\right)$ and $L\left(D,K\right)\;$ from polynomial interpolation. Right: Signal cumulants $\left(D,K\right)$ at fixed diffusion time colored by predictions of $D_{0}\;$and L as obtained with the *SigFit* approach, which relies on the estimation of $D_{0}$ and L via routine nonlinear least‐squares fitting on the MRI signal. The figure refers to the case when the minimum/maximum protocol *b*‐values are equal to b= 100/2000 s/mm^2^ and the diffusion gradient duration/separation is δ = 20 ms/Δ = 75 ms for SNR →∞ (no noise injected to the DW measurements) and in presence of IVIM contamination
**FIGURE S5** Examples of predictions of intrinsic cell diffusivity $D_{0}$ and cell size L on the validation set. (A–C) Scatter plots showing prediction of average intrinsic cell diffusivity $D_{0}$. (D–F) Scatter plots showing prediction of average cell size $D_{0}$. Left: Signal cumulants $\left(D,K\right)$ at fixed diffusion time colored by underlying ground truth $D_{0}$ and L. Middle: Signal cumulants $\left(D,K\right)$ at fixed diffusion time colored by predictions of $D_{0}$ and L as obtained with the *PolyMap* approach, which relies on using smooth functions $D_{0}\left(D,K\right)$ and $L\left(D,K\right)\;$from polynomial interpolation. Right: Signal cumulants $\left(D,K\right)$ at fixed diffusion time colored by predictions of $D_{0}$ and L as obtained with the *SigFit* approach, which relies on the estimation of $D_{0}$ and L via routine nonlinear least‐squares fitting on the MRI signal. The figure refers to the case when the minimum/maximum protocol *b*‐values are equal to b= 100/1000 s/mm^2^ and the diffusion gradient duration/separation is δ = 20 ms/Δ = 25 ms for SNR = 20 and in the presence of IVIM contamination
**FIGURE S6** *SigFit* prediction errors for $D_{0}$ and L scattered against ground‐truth values of $D_{0}$ and L for different diffusion gradient timings at a fixed maximum b‐value of 2000 s/mm^2^ and SNR = 20. From left to right: Different gradient timings (δ/Δ = 20/25 ms in [A] and [F]; δ/Δ = 40/50 ms in [B] and [G]; δ/Δ = 20/50 ms in [C] and [H]; δ/Δ = 10/50 ms in [D] and [I]; and δ/Δ = 20/75 ms in [E] and [J]). Plots on top (A–E) refer to $D_{0}$; plots on the bottom (F–J) refer to L. For each fixed value of $D_{0}$ (on top, or L on the bottom), median errors with interquartile ranges for varying L (on top, or varying $D_{0}$ on the bottom) are also reported
**FIGURE S7** *PolyMap* and *SigFit* prediction errors for $D_{0}$ and L scattered against ground‐truth values of $D_{0}$ and L, for different diffusion gradient timings at a fixed maximum b‐value of 2000 s/mm^2^ and SNR →∞ (no noise injected to the data). Top (A‐J, rows one and two): *PolyMap* results (estimation from cumulants $\left(D,K\right)$ via smooth polynomial functions), with $D_{0}$ errors on row one and L errors on row two. Bottom (K‐T, rows three and four): *SigFit* results (direct fitting on the MRI signal), with $D_{0}$ errors on row three and L errors on row four. From left to right: Different gradient timings (δ/Δ = 20/25 ms in [A] and [F]; δ/Δ = 40/50 ms in [B] and [G]; δ/Δ = 20/50 ms in [C] and [H]; δ/Δ = 10/50 ms in [D] and [I]; and δ/Δ = 20/75 ms in [E] and [J]). In rows one and three, median errors with interquartile ranges for varying L and fixed $D_{0}$ are reported. In rows two and four, median errors with interquartile ranges for varying $D_{0}$ and fixed L are reported
**FIGURE S8** *PolyMap* and *SigFit* prediction errors for $D_{0}$ and L scattered against ground‐truth values of $D_{0}$ and L for different diffusion gradient timings at a fixed maximum b‐value of 2000 s/mm^2^ and SNR = 20. Top (A‐J, rows one and two): *PolyMap* results (estimation from cumulants $\left(D,K\right)$ via smooth polynomial functions), with $D_{0}$ errors on row one and L errors on row two. Bottom (K‐T, rows three and four): *SigFit* results (direct fitting on the MRI signal), with $D_{0}$ errors on row three and L errors on row four. From left to right: Different gradient timings (δ/Δ = 20/25 ms in [A] and [F]; δ/Δ = 40/50 ms in [B] and [G]; δ/Δ = 20/50 ms in [C] and [H]; δ/Δ = 10/50 ms in [D] and [I]; and δ/Δ = 20/75 ms in [E] and [J]). In rows one and three, median errors with interquartile ranges for varying L and fixed $D_{0}$ are reported. In rows two and four, median errors with interquartile ranges for varying $D_{0}$ and fixed L are reported
**FIGURE S9** Examples of DW images obtained ex vivo on the two fixed mouse livers. (A) Images from the wild‐type (WT) liver, alongside image predictions based on fitted model parameters for *PolyMap* and *SigFit*. (B) Similar information as in (A) but for the patient‐derived xenograft (PDX) liver. (C,D) Examples of MRI measurements (i.e., logarithm of measured signals) from one representative voxel alongside *PolyMap* and *SigFit* fittings for the WT (C, left) and PDX (D, right) livers
**FIGURE S10** *SigFit* cell size map L in the two fixed liver samples scanned at 9.4 T: WT (top) and PDX (bottom). From left to right: Full *SigFit* estimation (cell size L and cell diffusivity $D_{0}$ are estimated jointly at fixed diffusion time); L estimation when $D_{0}$ is fixed and not estimated (values used for $D_{0}$: 0.5, 0.75, 1.0, 1.25, 1.25, and 1.5 μm^2^/ms, as shown from left to right). Median values of L across the entire samples are reported for each specimen and *SigFit* configuration
**FIGURE S11** Image patches illustrating the different microstructural environments observed in the two fixed mouse livers studied in this paper. Top: Patches from the WT liver, showing healthy hepatocytes surrounded by stellate cells and sinusoidal capillaries. Bottom: Patches from the PDX liver. On visual inspection, hepatocytes in the PDX appear to contain less fat than in the WT. Moreover, the PDX liver is characterized by a nonspecific, lymphoma‐like process, in which cells that are much smaller than hepatocytes invade vascular and extravascular spaces
**FIGURE S12** Investigation on the impact of the number of gradient directions used to compute directionally averaged signals. The figure shows results obtained for fitting performed on seven nonzero b‐values in the range $\left[100\frac{s}{{\mathrm{mm}}^{2}};\;2000\frac{s}{{\mathrm{mm}}^{2}}\right]$; δ = 20 ms, Δ = 75 ms; intrinsic cell diffusivity and cell size $\left(D_{0},\;L\right)\;\in \;\left[2.20\frac{\mu m^{2}}{\mathrm{ms}};\;2.40\frac{\mu m^{2}}{\mathrm{ms}}\right]\;\times \;\left[11\;\mu m;\;17\;\mu m\right]$; {3, 9, 21, 30, 61} isotropically distributed gradient directions for each nonzero b‐value; no IVIM contamination. (A) Directionally averaged DW signals obtained at SNR →∞for {3, 9, 21, 30, 61} gradient directions. (B,D) Distribution of apparent diffusion coefficient D and apparent kurtosis coefficient K calculated by fitting Equation 3 to directionally averaged signals over 1000 random noise instantiations with 20 ≤ SNR ≤ 100, for {3, 9, 21, 30, 61} gradient directions per b‐value. C,E, Distribution of diffusion tensor mean diffusivity MD and kurtosis tensor mean kurtosis MK calculated by fitting a full diffusion kurtosis tensor representation to all measurements over 1000 random noise instantiations with 20 ≤ SNR ≤ 100, for {3, 9, 21, 30, 61} gradient directions per b‐value. For full kurtosis tensor fitting we used the freely available DiPy package (https://dipy.org/documentation/1.4.1./examples_built/reconst_dki/#example‐reconst‐dki). Gradient directions were generated according to Cauryer et al (Magn Res Med. 2013; doi: 10.1002/mrm.24736; free download from http://www.emmanuelcaruyer.com/q‐space‐sampling.php)
**FIGURE S13** Investigation on the impact of the number of b‐values used for apparent diffusion coefficient D and apparent kurtosis coefficient K computation from Equation 3. The figure shows results obtained for {19, 10, 7, 4, 3} nonzero b‐values and δ = 20 ms, Δ = 75 ms; maximum b‐values of 1000 s/mm^2^ and 2000 s/mm^2^; $\left(D_{0},\;L\right)\;\in \;\left[2.20\frac{\mu m^{2}}{\mathrm{ms}};\;2.40\frac{\mu m^{2}}{\mathrm{ms}}\right]\;\times \;\left[11\;\mu m;\;17\;\mu m\right]$; no IVIM contamination. (A,C) Distribution of ADC D over 1000 random noise instantiations with 20 ≤SNR ≤ 100, {19, 10, 7, 4, 3} nonzero b‐values, maximum b‐value of 1000 s/mm^2^ (A), and 2000 s/mm^2^ (C). Each plot also indicates the value of D obtained with 19 nonzero b‐values and SNR →∞ for reference. (B,D) Distribution of apparent kurtosis coefficient K over 1000 random noise instantiations with 20 ≤ SNR ≤100, {19, 10, 7, 4, 3} nonzero b‐values, maximum b‐value of 1000 s/mm^2^ (B), and 2000 s/mm^2^ (D). Each plot also indicates the value of K obtained with 19 nonzero b‐values and SNR →∞ for reference
**TABLE S1** Median and interquartile range (within brackets) of histology‐derived cell‐size index $L_{\mathrm{histo}}$ and MRI metrics from the *PolyMap* and *SigFit* estimation procedures investigated in this work (average cell size L and average intracellular diffusivity $D_{0}$)

## Data Availability

The synthetic hepatocyte meshes and the code written to perform simulation and analyze the ex vivo MRI and histology data are made freely available online following publication ( https://github.com/fragrussu/MChepato ). Researchers interested in accessing the ex vivo mouse‐liver MRI and histology data can contact the corresponding author to stipulate relevant research and data transfer agreements.
