# Supplementary material for: Diffusion MRI signal cumulants and hepatocyte microstructure at fixed diffusion time: Insights from simulations, 9.4T imaging, and histology
Source: Magn Reson Med. 2022 Feb 18;88(1):365–79. doi: 10.1002/mrm.29174 (PMC9303340; doi:10.1002/mrm.29174)
Supplement: Supplementary file 1 — FIGURE S1 Synthetic hepatocytes used in this study for Monte Carlo simulations (water diffusion was simulated within such synthetic cells), obtained by perturbing the position of the vertices of triangularly meshed regular prisms. Top to bottom: Different shapes of the prism bases (square, pentagonal, hexagonal). Left to right: Different unique perturbations. The figure also illustrates L, which is equal to the base‐to‐base height as well as the diameter of the circumcircle relative to each base FIGURE S2 Scatter plots of (D,K) color‐coded by the underlying average intrinsic cell diffusivity D0 (A–E) and cell size L (F–J), as obtained when no noise is added to the synthetic MRI signals. From left to right: Different diffusion times (δ/Δ = 20/25 ms in [A] and [F]; δ/Δ = 40/50 ms in [B] and [G]; δ/Δ = 20/50 ms in [C] and [H]; δ/Δ = 10/50 ms in [D] and [I]; and δ/Δ = 20/75 ms in [E] and [J]). The figure refers to a minimum/maximum protocol b‐value of b = 100/2000 s/mm2. Noise‐free intracellular diffusion‐weighted (DW) signals are contaminated by intravoxel incoherent motion (IVIM)–like partial volume FIGURE S3 Scatter plots of (D,K) color‐coded by the underlying average intrinsic cell diffusivity D0 (A–E) and cell size L (F–J), as obtained when noise is added to the synthetic MRI signals at an SNR at b = 0 of 20. From left to right: Different diffusion times (δ/Δ = 20/25 ms in [A] and [F]; δ/Δ = 40/50 ms in [B] and [G]; δ/Δ = 20/50 ms in [C] and [H]; δ/Δ = 10/50 ms in [D] and [I]; and δ/Δ = 20/75 ms in [E] and [J]). The figure refers to a minimum/maximum protocol b‐value of b = 100/1000 s/mm2. Noise‐free intracellular DW signals are contaminated by IVIM‐like partial volume FIGURE S4 Examples of predictions of intrinsic cell diffusivity D0 and cell size L on the validation set. (A–C) Scatter plots showing prediction of average intrinsic cell diffusivity D0. (D–F) Scatter plots showing prediction of average cell size D0. Left: Signal cumulants D,K at fixed diffusion time [file MRM-88-365-s001.pdf]

Supporting Information Figure S1

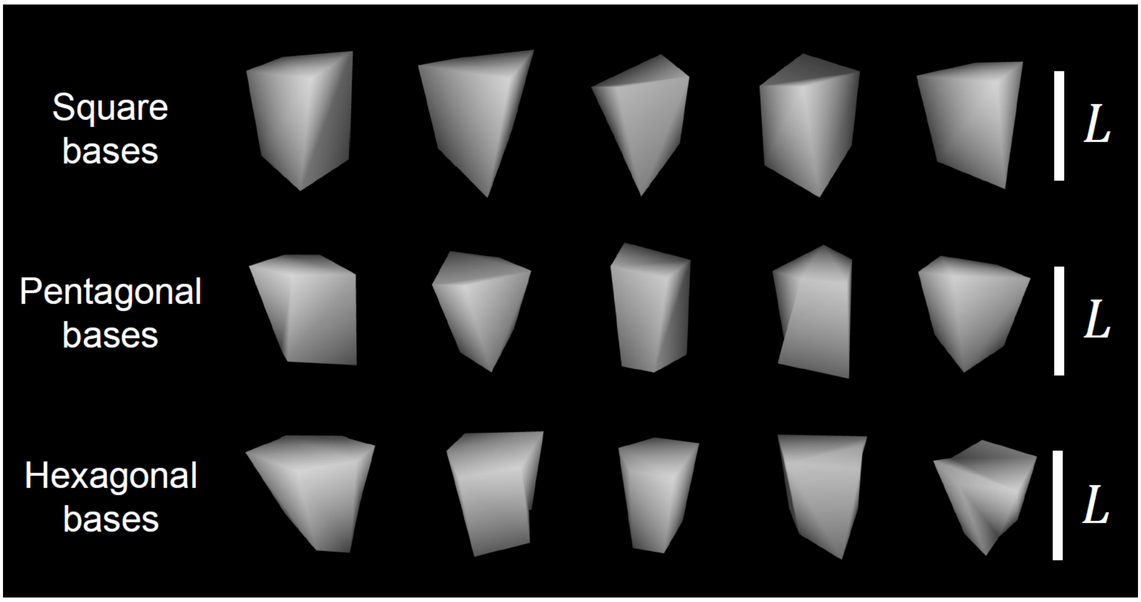

FIGURE S1 Synthetic hepatocytes used in this study for Monte Carlo simulations (water diffusion was simulated within such synthetic cells), obtained by perturbing the position of the vertices of triangularly-meshed regular prisms. Top to bottom: different shapes of the prism bases (square, pentagonal, hexagonal). Left to right: different unique perturbations. The figure also illustrates L, which is equal to the base-to-base height as well as the diameter of the circumcircle relative to each base.

Supporting Information Figure S2

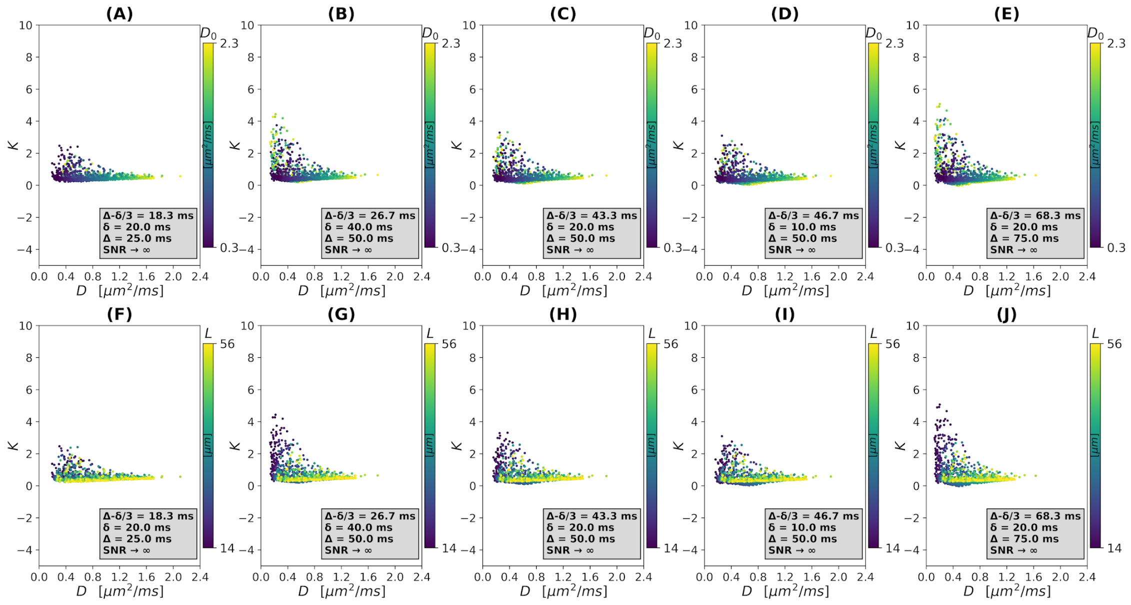

FIGURE S2 Scatter plots of (D, K) colour-coded by the underlying average intrinsic cell diffusivity D_0 (top, A-E) and cell size L (bottom, F-J), as obtained when no noise is added to the synthetic MRI signals. From left to right: different diffusion times (δ/Δ = 20/25 ms in A, F; δ/Δ = 40/50 ms in B, G; δ/Δ = 20/50 ms in C, H; δ/Δ = 10/50 ms in D, I; δ/Δ = 20/75 ms in E, J). The figure refers to a minimum/maximum protocol b-value of b = 100/2000 s/mm^2. Noise-free intra-cellular DW signals are contaminated by IVIM-like partial volume.

Supporting Information Figure S3

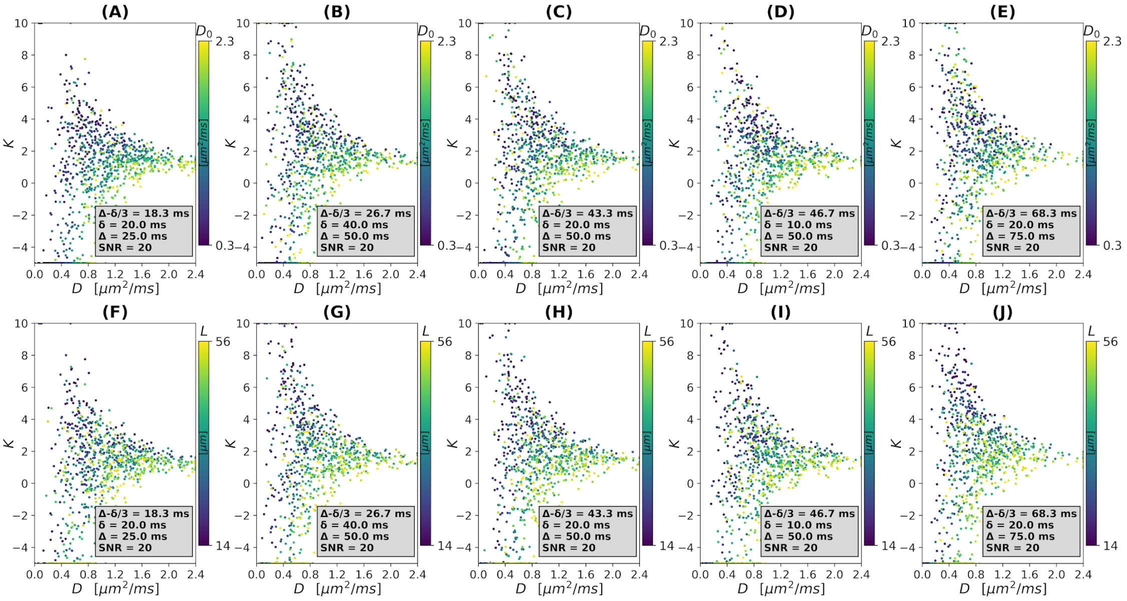

FIGURE S3 Scatter plots of (D, K) colour-coded by the underlying average intrinsic cell diffusivity D_0 (top, A-E) and cell size L (bottom, F-J), as obtained when noise is added to the synthetic MRI signals at an SNR at b = 0 of 20. From left to right: different diffusion times (δ/Δ = 20/25 ms in A, F; δ/Δ = 40/50 ms in B, G; δ/Δ = 20/50 ms in C, H; δ/Δ = 10/50 ms in D, I; δ/Δ = 20/75 ms in E, J). The figure refers to a minimum/maximum protocol b-value of b = 100/1000 s/mm^2. Noise-free intra-cellular DW signals are contaminated by IVIM-like partial volume.

Supporting Information Figure S4

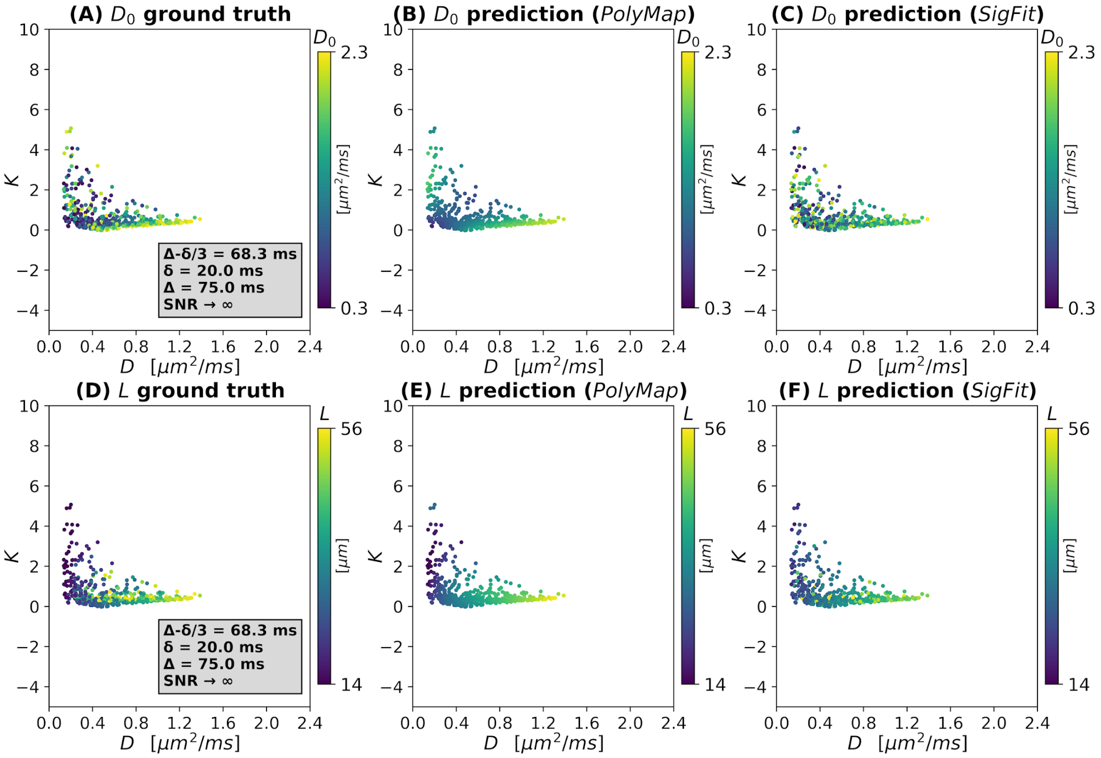

FIGURE S4 Examples of predictions of intrinsic cell diffusivity D_0 and cell size L on the validation set from. Top: scatter plots showing prediction of average intrinsic cell diffusivity D_0 (panels A to C); bottom: scatter plots showing prediction of average cell size L (panels D to F). Left: signal cumulants (D, K) at fixed diffusion time coloured by underlying ground truth D_0 and L. Middle: signal cumulants (D, K) at fixed diffusion time coloured by predictions of D_0 and L as obtained with the *PolyMap* approach, which relies on using smooth functions $D_0(D, K)$ and $L(D, K)$ from polynomial interpolation. Right: signal cumulants (D, K) at fixed diffusion time coloured by predictions of D_0 and L as obtained with the *SigFit* approach, which relies on the estimation of D_0 and L via routine non-linear least square fitting on the MRI signal. The figure refers to the case when the minimum/maximum protocol *b*-values is equal to b = 100/2000 s/mm^2 and the diffusion gradient duration/separation is δ = 20ms/Δ = 75 ms, for SNR → ∞ (no noise injected to the DW measurements) and in presence of IVIM contamination.

Supporting Information Figure S5

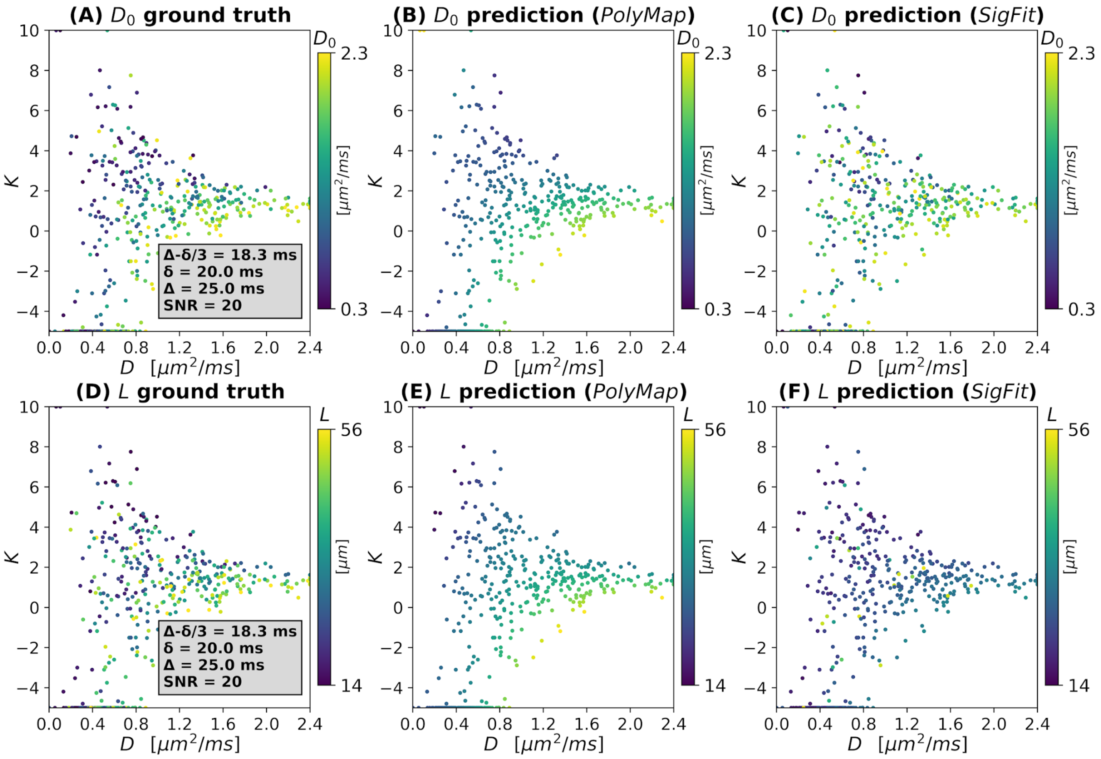

FIGURE S5 Examples of predictions of intrinsic cell diffusivity D_0 and cell size L on the validation set from. Top: scatter plots showing prediction of average intrinsic cell diffusivity D_0 (panels A to C); bottom: scatter plots showing prediction of average cell size L (panels D to F). Left: signal cumulants (D, K) at fixed diffusion time coloured by underlying ground truth D_0 and L. Middle: signal cumulants (D, K) at fixed diffusion time coloured by predictions of D_0 and L as obtained with the *PolyMap* approach, which relies on using smooth functions $D_0(D, K)$ and $L(D, K)$ from polynomial interpolation. Right: signal cumulants (D, K) at fixed diffusion time coloured by predictions of D_0 and L as obtained with the *SigFit* approach, which relies on the estimation of D_0 and L via routine non-linear least square fitting on the MRI signal. The figure refers to the case when the minimum/maximum protocol *b*-values is equal to b = 100/1000 s/mm^2 and the diffusion gradient duration/separation is δ = 20ms/Δ = 25 ms, for SNR of 20 (no noise injected to the DW measurements) and in presence of IVIM contamination.

Supporting Information Figure S6

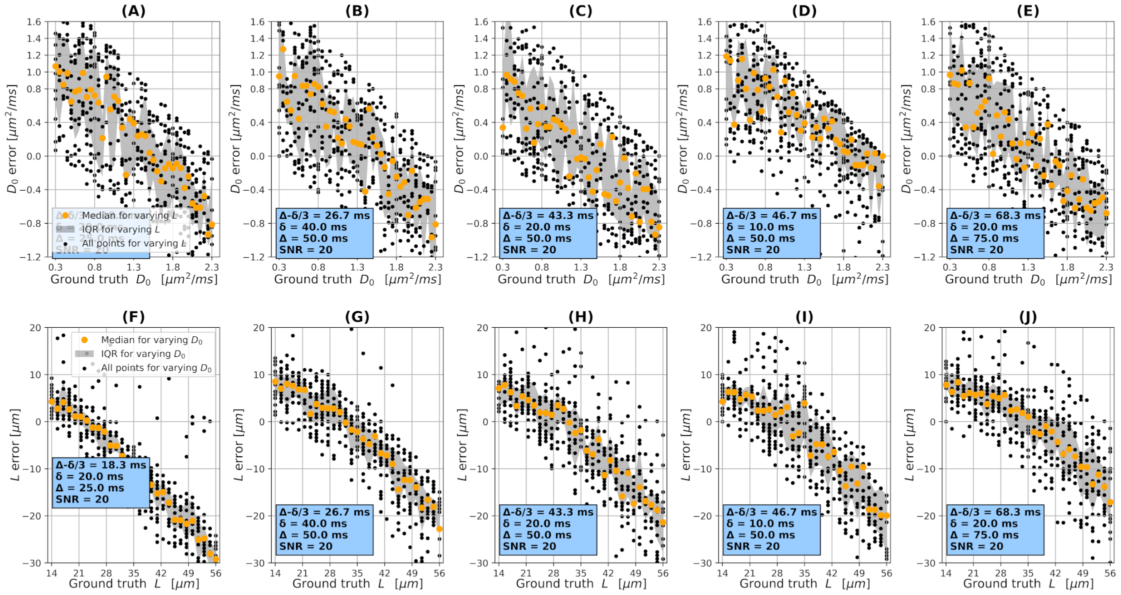

FIGURE S6 *SigFit* prediction errors for D_0 and L scattered against ground truth values of D_0 and L, for different diffusion gradient timings at a fixed maximum b-value of 2000 s/mm^2 and SNR = 20. From left to right: different gradient timings (δ/Δ = 20/25 ms in A, F; δ/Δ = 40/50 ms in B, G; δ/Δ = 20/50 ms in C, H; δ/Δ = 10/50 ms in D, I; δ/Δ = 20/75 ms in E, J). Plots on top (A-E) refer to D_0; plots to the bottom (F-J) refer to L. For each fixed value of D_0 (on top, or L to the bottom), median errors with interquartile ranges for varying L (on top, or varying D_0 to the bottom) are also reported.

Supporting Information Figure S7

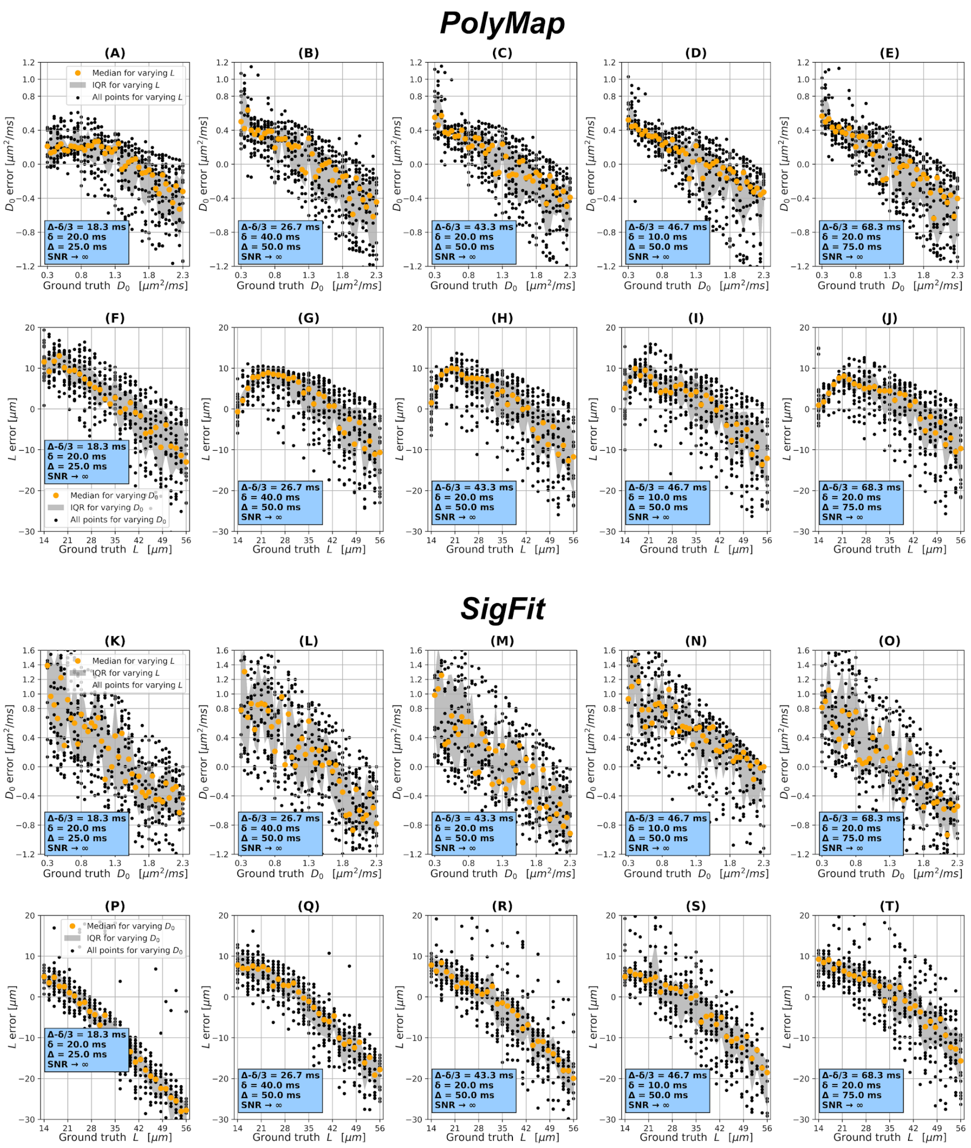

FIGURE S7 *PolyMap* and *SigFit* prediction errors for D_0 and L scattered against ground truth values of D_0 and L, for different diffusion gradient timings at a fixed maximum b-value of 2000 s/mm^2 and SNR →∞ (no noise injected to the data). Top (panels A to J, rows one and two): *PolyMap* results (estimation from cumulants (D, K) via smooth polynomial functions), with D_0 errors on row one and L errors on row two. Bottom (panels K to T, rows three and four): *SigFit* results (direct fitting on the MRI signal), with D_0 errors on row three and L errors on row four. From left to right: different gradient timings (δ/Δ = 20/25 ms in A, F; δ/Δ = 40/50 ms in B, G; δ/Δ = 20/50 ms in C, H; δ/Δ = 10/50 ms in D, I; δ/Δ = 20/75 ms in E, J). In rows one and three, median errors with interquartile ranges for varying L and fixed D_0 are reported. In rows two and four, median errors with interquartile ranges for varying D_0 and fixed L are reported.

Supporting Information Figure S8

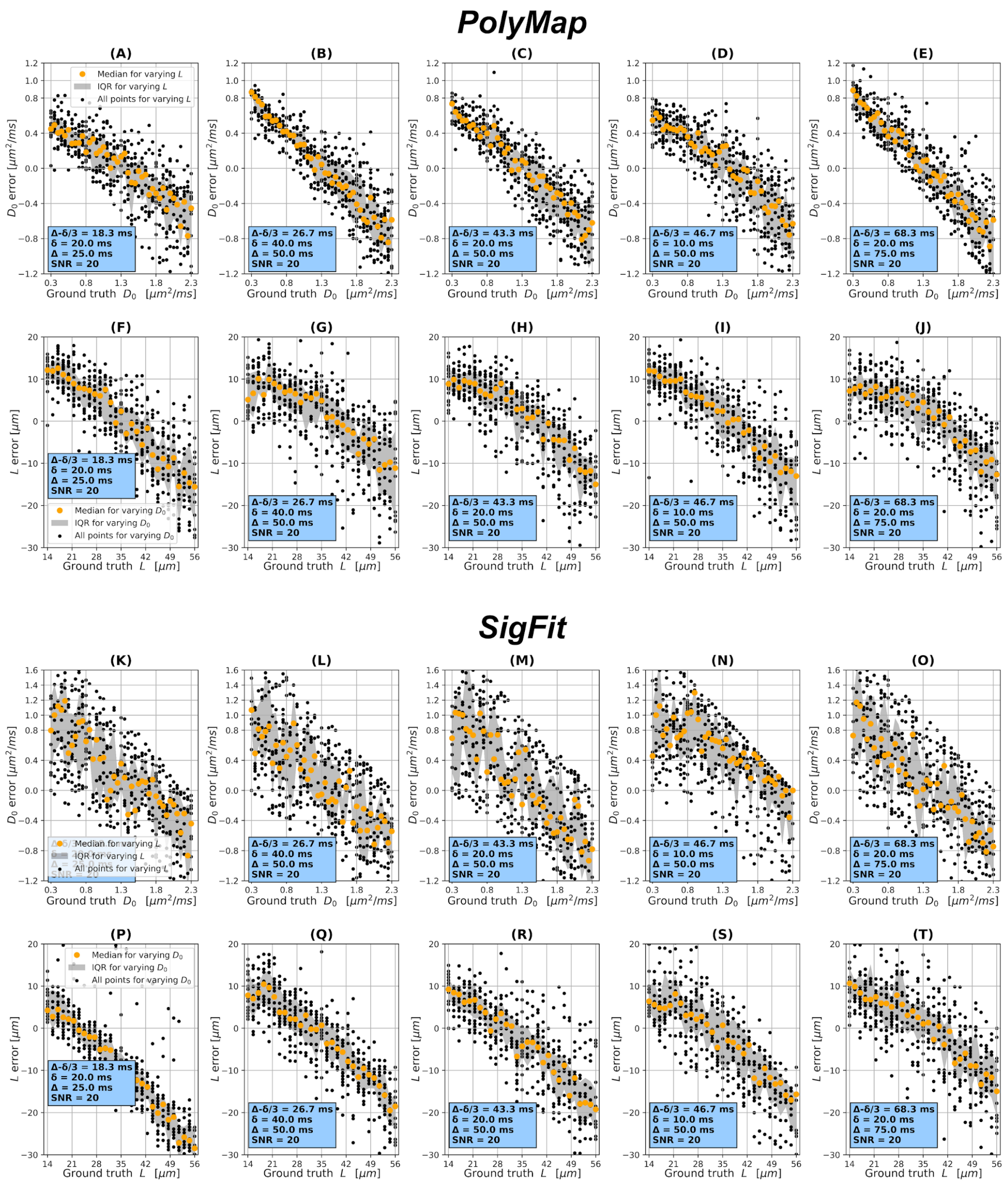

FIGURE S8 *PolyMap* and *SigFit* prediction errors for D_0 and L scattered against ground truth values of D_0 and L, for different diffusion gradient timings at a fixed maximum b-value of 1000 s/mm^2 and SNR = 20. Top (panels A to J, rows one and two): *PolyMap* results (estimation from cumulants (D, K) via smooth polynomial functions), with D_0 errors on row one and L errors on row two. Bottom (panels K to T, rows three and four): *SigFit* results (direct fitting on the MRI signal), with D_0 errors on row three and L errors on row four. From left to right: different gradient timings (δ/Δ = 20/25 ms in A, F; δ/Δ = 40/50 ms in B, G; δ/Δ = 20/50 ms in C, H; δ/Δ = 10/50 ms in D, I; δ/Δ = 20/75 ms in E, J). In rows one and three, median errors with interquartile ranges for varying L and fixed D_0 are reported. In rows two and four, median errors with interquartile ranges for varying D_0 and fixed L are reported.

Supporting Information Figure S9

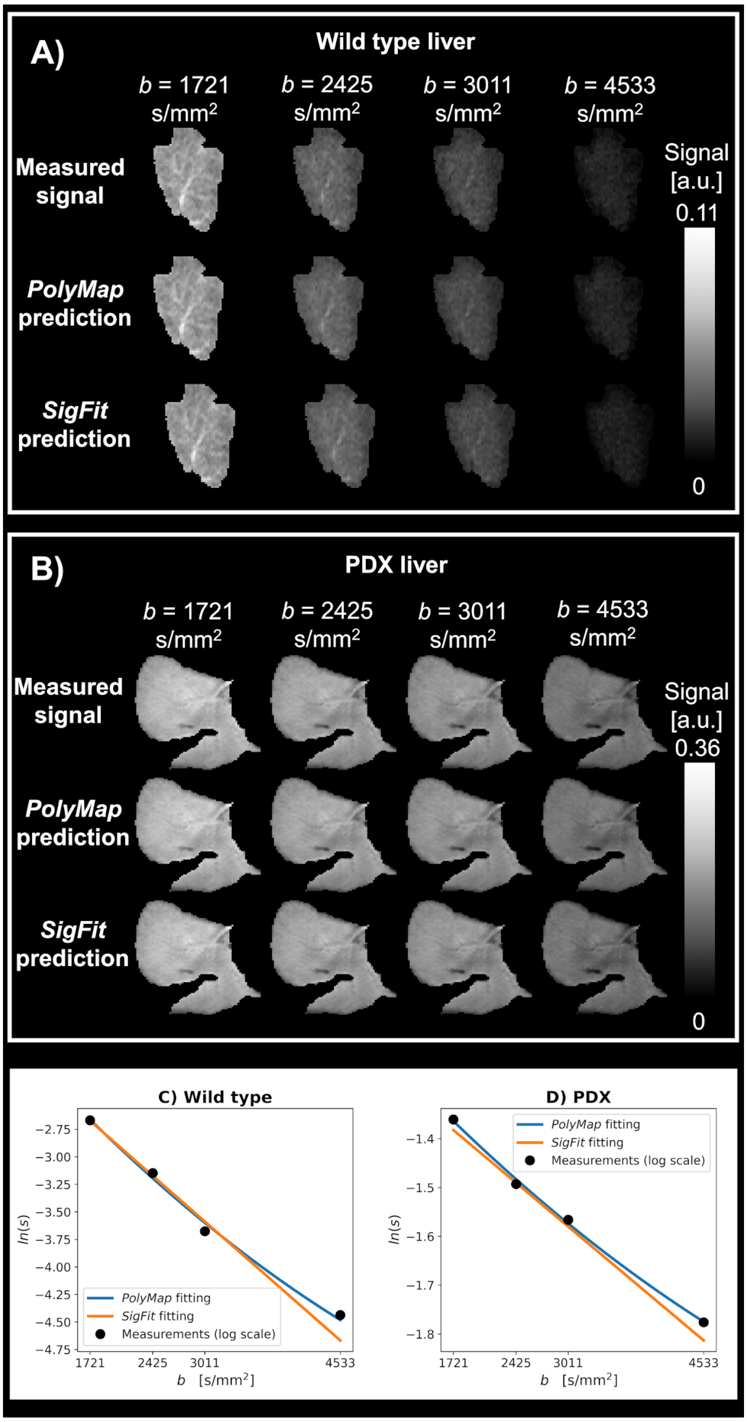

FIGURE S9 Examples of DW images obtained *ex vivo* on the two fixed mouse livers. A (top): images from the wild type (WT) liver, alongside image predictions based on fitted model parameters for *PolyMap* and *SigFit*. B: similar information as in A but for the patient-derived xenograft (PDX) liver. C and D: examples of MRI measurements (i.e., logarithm of measured signals) from one representative voxel alongside *PolyMap* and *SigFit* fittings for the WT (C, left) and PDX (D, right) livers.

Supporting Information Figure S10

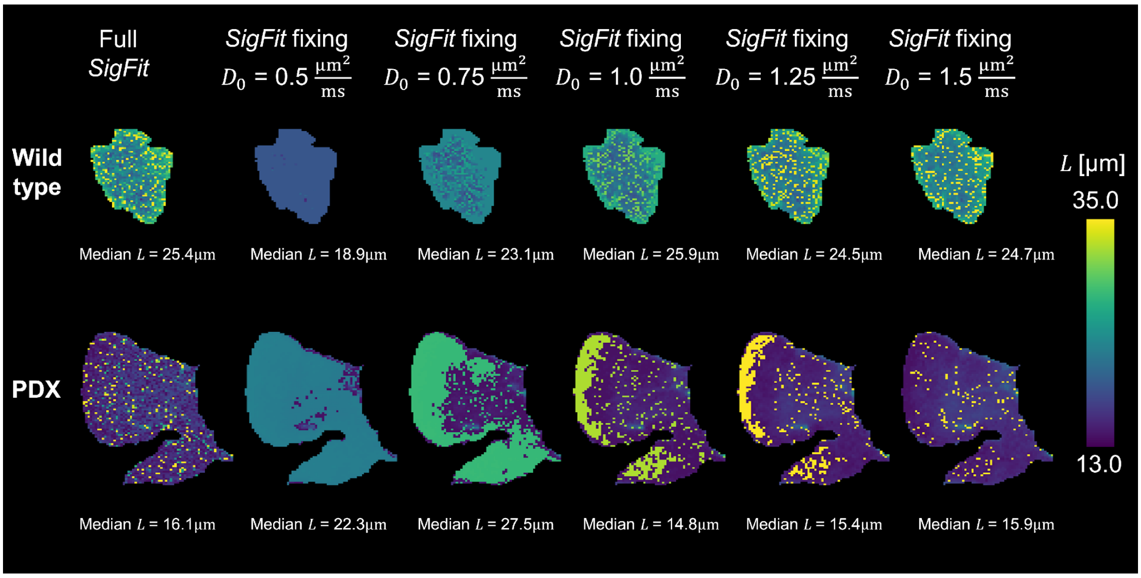

FIGURE S10 *SigFit* cell size map L in the two fixed liver samples scanned at 9.4T. Top: wild type (WT); bottom: patient-derived xenograft (PDX). From left to right: full *SigFit* estimation (cell size L and cell diffusivity D_0 are estimated jointly at fixed diffusion time); L estimation when D_0 is fixed, and not estimated (values used for D_0: 0.5, 0.75, 1.0, 1.25, 1.25; 1.5 μm^2/ms, as shown from left to right). Median values of L across the entire samples are reported for each specimen and *SigFit* configuration.

Supporting Information Figure S11

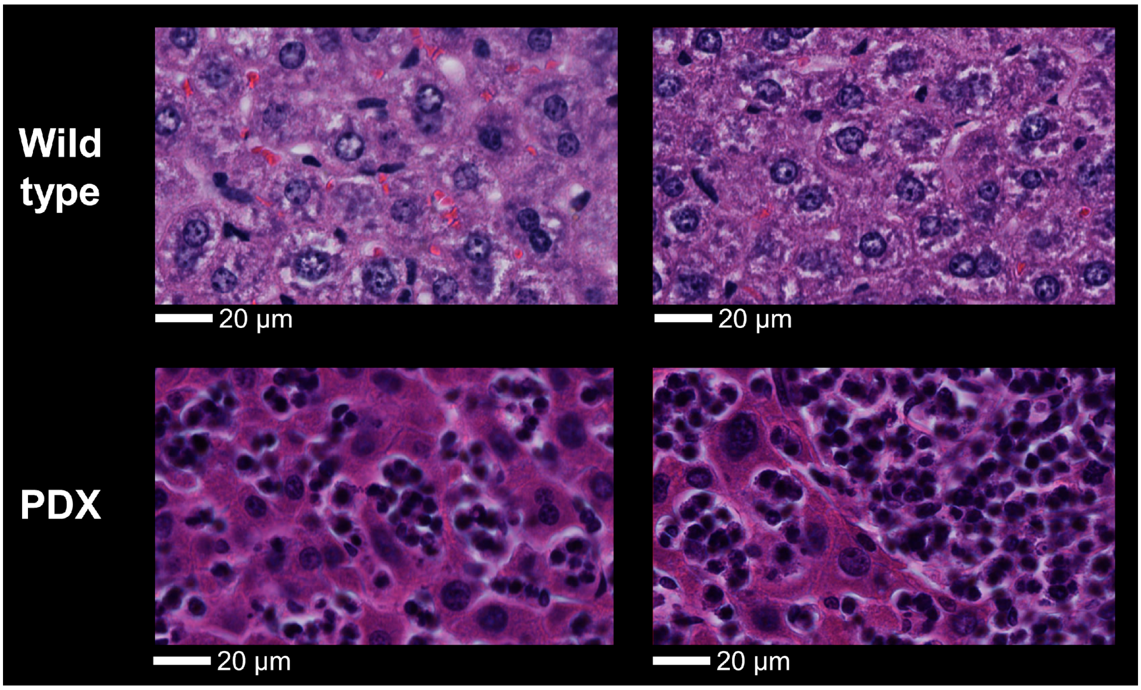

FIGURE S11 Image patches illustrating the different microstructural environments observed in the two fixed mouse livers studied in this paper. Top: patches from the wild type (WT) liver, showing healthy hepatocytes surrounded by stellate cells and sinusoidal capillaries. Bottom: patches from the patient-derived xenograft (PDX) liver. On visual inspection, hepatocytes in the PDX appear to contain less fat than in the WT. Moreover, the PDX liver is characterised by a non-specific, lymphoma-like process, in which cells that are much smaller than hepatocytes invade vascular and extra-vascular spaces.

Supporting Information Figure S12

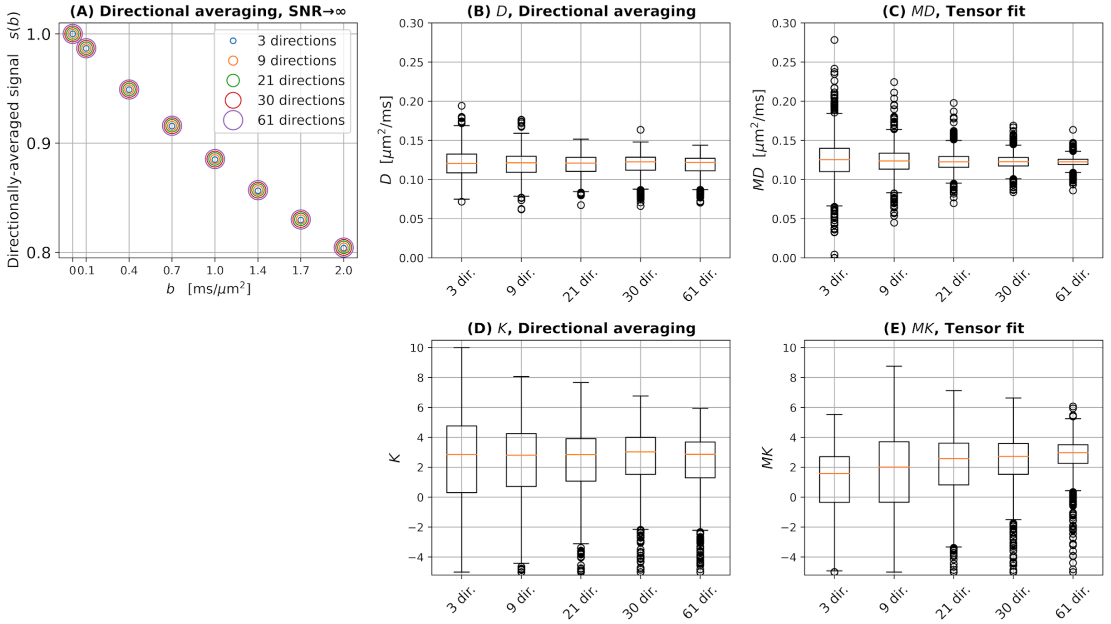

FIGURE S12 Investigation on the impact of the number of gradient directions used to compute directionally-averaged signals. The figure shows results obtained for fitting performed on 7 non-zero b-values in the range $\left[100\frac{s}{mm^2};\ 2000\frac{s}{mm^2}\right]$; δ = 20ms, Δ = 75 ms; intrinsic cell diffusivity and cell size $\left(D_0,\ L\right) \in \left[2.20\frac{\mu m^2}{ms};\ 2.40\frac{\mu m^2}{ms}\right] \times [11\ \mu m;\ 17\ \mu m]$; {3, 9, 21, 30, 61} isotropically-distributed gradient directions for each non-zero b-value; no IVIM contamination. (A): directionally-averaged DW signals obtained at SNR →∞ for {3, 9, 21, 30, 61} gradient directions. (B) and (D): distribution of apparent diffusion coefficient D and apparent kurtosis coefficient K calculated by fitting Equation 3 to directionally-averaged signals over 1000 random noise instantiations with 20 ≤ SNR ≤ 100, for {3, 9, 21, 30, 61} gradient directions per b-value. (C) and (E): Distribution of diffusion tensor mean diffusivity MD and kurtosis tensor mean kurtosis MK calculated by fitting a full diffusion kurtosis tensor representation to all measurements over 1000 random noise instantiations with 20 ≤ SNR ≤ 100, for {3, 9, 21, 30, 61} gradient directions per b-value. For full kurtosis tensor fitting we used the freely available `DiPy` package (https://dipy.org/documentation/1.4.1./examples_built/reconst_dki/#example-reconst-dki). Gradient directions were generated according to Cauryer E et al, Magn Res Med 2013, doi: 10.1002/mrm.24736 (free download from: http://www.emmanuelcaruyer.com/q-space-sampling.php).

Supporting Information Figure S13

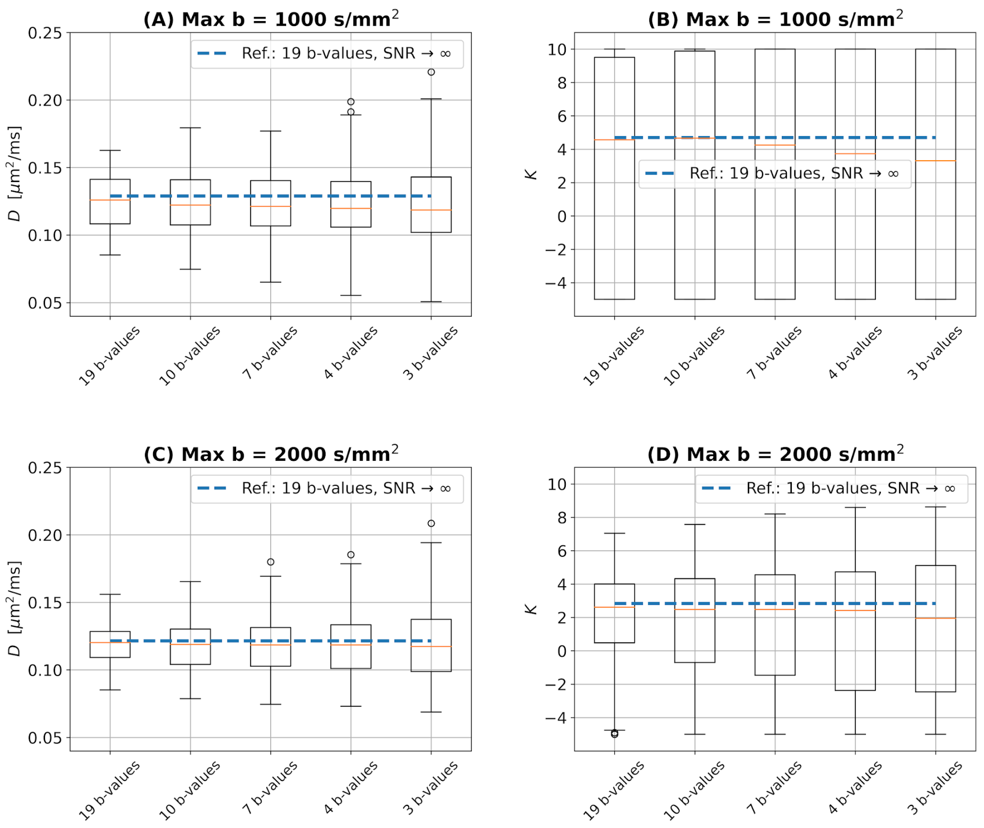

FIGURE S13 Investigation on the impact of the number of b-values used for apparent diffusion coefficient D and apparent kurtosis coefficient K computation from Equation 3. The figure shows results obtained for {19, 10, 7, 4, 3} non-zero b-values and δ = 20ms, Δ = 75 ms; maximum b-values of 1000 s/mm^2 and 2000 s/mm^2; $\left(D_0, L\right) \in \left[2.20\frac{\mu m^2}{ms};\ 2.40\frac{\mu m^2}{ms}\right] \times [11\ \mu m;\ 17\ \mu m]$; no IVIM contamination. (A) and (C): distribution of apparent diffusion coefficient D over 1000 random noise instantiations with 20 ≤ SNR ≤ 100, {19, 10, 7, 4, 3} non-zero b-values, maximum b-value of 1000 s/mm^2 (A) and 2000 s/mm^2 (C). Each plot also indicates the value of D obtained with 19 non-zero b-values and SNR →∞ for reference. (B) and (D): distribution of apparent kurtosis coefficient K over 1000 random noise instantiations with 20 ≤ SNR ≤ 100, {19, 10, 7, 4, 3} non-zero b-values, maximum b-value of 1000 s/mm^2 (B) and 2000 s/mm^2 (D). Each plot also indicates the value of K obtained with 19 non-zero b-values and SNR →∞ for reference.

Supporting Information Table S1

TABLE S1 Median and interquartile range (IQR, within brackets) of histology-derived cell size index L_{histo} and MRI metrics from the *PolyMap* and *SigFit* estimation procedures investigated in this work (average cell size L and average intra-cellular diffusivity D_0).

	**Histology**	**MRI**			
	L_{histo}	*PolyMap* L	*SigFit* L	*PolyMap* D_0	*SigFit* D_0
**Wild type**	20.9 (19.0; 23.8) μm	43.0 (37.4; 49.9) μm	25.4 (23.3; 29.0) μm	1.50 (1.38; 1.63) μm^2/ms	1.45 (1.08; 1.89) μm^2/ms
**PDX**	14.2 (13.2; 16.8) μm	18.1 (13.9; 22.3) μm	16.1 (15.4; 17.6) μm	0.79 (0.47; 0.97) μm^2/ms	1.55 (1.08; 1.92) μm^2/ms
